# GPU-accelerated connectome discovery at scale

**DOI:** 10.1038/s43588-022-00250-z

**Published:** 2022-05-30

**Authors:** Varsha Sreenivasan, Sawan Kumar, Franco Pestilli, Partha Talukdar, Devarajan Sridharan

**Affiliations:** 1 Centre for Neuroscience, Indian Institute of Science, Bangalore, India; 2 Department of Computational and Data Sciences, Indian Institute of Science, Bangalore, India; 3 Department of Psychology, University of Texas at Austin, Austin, TX USA; 4 Department of Computer Science and Automation, Indian Institute of Science, Bangalore, India

**Keywords:** Diffusion tensor imaging, Software, Cognitive neuroscience, Computational models

## Abstract

Diffusion magnetic resonance imaging and tractography enable the estimation of anatomical connectivity in the human brain, in vivo. Yet, without ground-truth validation, different tractography algorithms can yield widely varying connectivity estimates. Although streamline pruning techniques mitigate this challenge, slow compute times preclude their use in big-data applications. We present ‘Regularized, Accelerated, Linear Fascicle Evaluation’ (ReAl-LiFE), a GPU-based implementation of a state-of-the-art streamline pruning algorithm (LiFE), which achieves >100× speedups over previous CPU-based implementations. Leveraging these speedups, we overcome key limitations with LiFE’s algorithm to generate sparser and more accurate connectomes. We showcase ReAl-LiFE’s ability to estimate connections with superlative test–retest reliability, while outperforming competing approaches. Moreover, we predicted inter-individual variations in multiple cognitive scores with ReAl-LiFE connectome features. We propose ReAl-LiFE as a timely tool, surpassing the state of the art, for accurate discovery of individualized brain connectomes at scale. Finally, our GPU-accelerated implementation of a popular non-negative least-squares optimization algorithm is widely applicable to many real-world problems.

## Abstract (web summary)

Accurate structural brain connectivity estimation is key to uncovering brain–behavior relationships. ReAl-LiFE, a GPU-accelerated approach, is applied for fast and reliable evaluation of individualized brain connectomes at scale.

## Main

Intact anatomical connectivity among brain areas is critical to cognition^1^. Accurate estimation of anatomical connections in vivo is critical not only for uncovering the neural underpinnings of human behavior, but also for understanding the genetic bases of neurological disorders^2^.

Diffusion magnetic resonance imaging (dMRI), followed by tractography, enables the estimation of anatomical connectivity in the human brain, in vivo^3^. dMRI measures the diffusion of water molecules in the brain’s white matter, then tractography algorithms estimate axonal structures based on restricted diffusion, post hoc^4^. However, dMRI and tractography algorithms are prone to challenges such as acquisition noise and redundant fiber geometries. As part of an international tractography challenge, a recent study^5^ compiled efforts by 20 teams that estimated a whole-brain connectome from a simulated dMRI scan, which was, in turn, generated with simulated ‘ground-truth’ fiber bundles. The diverse success rates across the different teams—in terms of their ability to match the ground truth—underscores the magnitude of this challenge. Because actual ground-truth connectivity in the brain is typically unavailable in vivo, direct validation of tractography in the human brain remains elusive.

Streamline pruning and evaluation algorithms represent a state-of-the-art, post-processing approach to address these challenges^3,6,7^. Linear Fascicle Evaluation (LiFE) is a recent, state-of-the-art model that prunes out spurious fibers based on the quality of fit to the underlying diffusion signal^3^. Yet, LiFE’s algorithm is implemented on central processing units (CPUs) and suffers from both speed and memory bottlenecks^3^, which preclude its application for connectome evaluation at scale.

In this Brief Communication, we present an improvement to LiFE—Regularized, Accelerated, Linear Fascicle Evaluation, or ReAl-LiFE—for rapid and accurate connectome evaluation at scale. We improve the LiFE algorithm by introducing an explicit regularization (sparsity) penalty into its objective function, and present a scalable graphics processing unit (GPU) implementation that routinely achieves orders of magnitude (>100×) speedups over CPU implementations while also estimating more sparse and more consistent connectomes. Next, we show that ReAl-LiFE performs at par with, and even outperforms, other state-of-the-art approaches (for example, SIFT2^6^ and COMMIT2^8^) in terms of estimating streamlines with high test–retest reliability. Finally, we apply ReAl-LiFE to identify structural connectivity correlates of behavior in a cohort of 200 participants drawn from the Human Connectome Project (HCP) database^9^. We propose ReAl-LiFE as an effective tool, surpassing the state of the art, for rapid and accurate connectome discovery at scale.

**Fig. 1:**
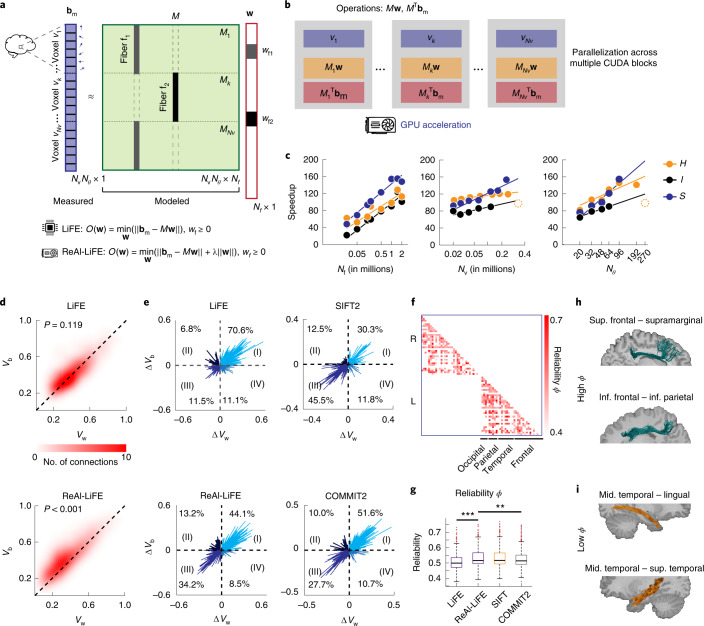
Rapid and reliable connectome evaluation with the ReAl-LiFE algorithm. **a**, Schematic of the LiFE model. *O*(**w**), objective function value. **b**, Schematic of the GPU-accelerated LiFE algorithm. **c**, Left: speedups as a function of the number of fibers *N*_f_ for datasets *H* (yellow), *I* (black) and *S* (blue). The *x* axes are shown in log scale. Middle: speedups as a function of the number of voxels *N*_v_ in each diffusion MRI volume. Right: speedups as a function of the number of gradient directions *N*_*θ*_ for each diffusion MRI scan. Filled circles, connectome sizes tested. Solid lines, logarithmic fit. Dashed open circles indicate points excluded from the logarithmic fit. **d**, Heatmaps showing within-participant variability (*V*_w_) against between-participant variability (*V*_b_) across *n* = 561 connections from five participants’ test–retest data, following pruning with the LiFE algorithm (top) and the ReAl-LiFE algorithm (bottom). Dashed diagonal line, line of equality. One-sided Wilcoxon signed-rank test, *P* = 0.119 (top), ***P < 0.001 (bottom). **e**, Vector plots demonstrating the improvement in reliability, relative to the unpruned connectome, following pruning with each of four different methods. Each plot shows change in within-participant variability (Δ*V*_w_) against change in between-participant variability (Δ*V*_b_) following pruning with LiFE (top left), ReAl-LiFE (bottom left), SIFT2 (top right) and COMMIT2 (bottom right). Each vector corresponds to one of the 561 connections evaluated. **f**, Reliability matrix, whose (*i*, *j*)th entry indicates the reliability measure (*ϕ* = *V*_b_/(*V*_b_ + *V*_w_)) of each pair of (*n* = 561) intra-hemispheric connections between regions *i* and *j*. Deeper shades indicate connections with higher test–retest reliability. R, right; L, left. **g**, Average reliability (*ϕ*) across all connections (*n* = 561) after pruning with LiFE, ReAl-LiFE, SIFT2 and COMMIT2. Center line, median; box limits, upper and lower quartiles; whiskers, 1.5× interquartile range; points, outliers. One-sided Wilcoxon signed-rank test: LiFE versus ReAl-LiFE, ***P < 0.001; ReAl-LiFE versus COMMIT2, **P < 0.01. **h**, Exemplar tracts corresponding to two connections with high consistency. **i**, Exemplar tracts corresponding to two connections with low consistency. Source data

We introduced a preliminary version of the ReAl-LiFE algorithm in an earlier study^10^ (Fig. 1a,b); this implementation achieved 50–100× speedups over CPU implementations of LiFE. In the present study, we optimize the algorithm further (Methods) to achieve even greater speedups (>100×, up to 155×; Methods). We demonstrate these speedups with three different diffusion MRI datasets.

We tested for speedups, first, with a state-of-the-art diffusion MRI dataset (dataset *H*; *N*_v_ = 437,495, *N*_*θ*_ = 270) from the HCP database^9^. We generated connectomes of seven different sizes, ranging from 50,000 to two million fibers (Methods). The streamlines in these connectomes were then pruned with the CPU implementation as well as with our GPU implementation of LiFE (Methods). The GPU implementation produced substantial speedups, ranging from 62-fold (62×; 95% confidence interval (CI), [59.8, 63.4]) for a connectome with 50,000 fibers to 129-fold (129×; 95% CI, [128.8, 129]) for a connectome with 1.5 million fibers (Fig. 1c). We evaluated these speedups on two other, independently acquired datasets: a dMRI dataset acquired in house (dataset *I*; *N*_v_ = 116,468, *N*_*θ*_ = 64; Methods) and a dataset used in the original LiFE study^3^ (dataset *S*; *N*_v_ = 247,969, *N*_*θ*_ = 96; Methods). Again, we observed maximum speedups of 124× (95% CI, [123.6, 124.4]; dataset *I*) and 155× (95% CI, [155.1, 155.2]; dataset *S*) for connectomes with 1.5 million fibers (Fig. 1c).

We also tested how speedups scaled with the number of voxels (*N*_v_) and number of diffusion directions (*N*_*θ*_). Overall, speedups scaled fastest with the number of diffusion directions, followed by the number of voxels (Supplementary Fig. 1). We also compared convergence times for ReAl-LiFE with two other pruning algorithms, SIFT^7^ and COMMIT2^8^. ReAl-LiFE performed comparably to SIFT and COMMIT2, with a slight advantage for our approach, especially at larger connectome sizes (Supplementary Fig. 1 and Supplementary Information).

Incorporating a sparsity-inducing prior (L1-norm of fiber weights; Methods) enabled ReAl-LiFE to generate sparser and more accurate connectomes. Yet, previous studies have indicated that such a sparsity-inducing prior may increase the chances of false negatives (missed fibers), particularly when duplicate fibers occur in the connectome^11^. We addressed this challenge precisely by constructing an artificial connectome comprised entirely of near-identical, duplicate streamlines, and show that pruning with ReAl-LiFE largely ameliorated this challenge (Supplementary Fig. 2). In addition, we show that ReAl-LiFE reduced overfitting and produced more consistent connectomes, as compared to LiFE (Supplementary Figs. 3 and 4).

We next quantified the reliability of the ReAl-LiFE algorithm, comparing it with that of LiFE, SIFT2^6^ and COMMIT2^8^. We performed a test–retest reliability analysis^12^ using a sample of *n* = 5 participants drawn from the HCP database (Methods). For each participant, pairwise intra-hemispheric connectivity was computed, the total variability of which was partitioned into between-participant (*V*_b_) and within-participant (*V*_w_) components (Methods).

Following pruning with LiFE, within- and between-participant variabilities were comparable (*V*_b_, CI [0.411, 0.435]; *V*_w_, CI [0.407, 0.432]; *P* = 0.119; effect size = 0.025; Fig. 1d), but, following ReAl-LiFE pruning, within-participant variability was significantly lower than between-participant variability (*V*_b_, CI [0.374, 0.401]; *V*_w_, CI [0.337, 0.365]; *P* < 0.001, effect size = 0.217; Fig. 1d). Thus, ReAl-LiFE pruning increased test–retest reliability of the estimated connection weights. Moreover, between-participant variability following ReAl-LiFE pruning was significantly lower than that following LiFE pruning (*P* < 0.001).

We further quantified the test–retest reliability with a reliability index (*ϕ*; Methods). A greater reliability index is a hallmark of efficient pruning. Compared with LiFE, pruning with ReAl-LiFE yielded a significantly higher reliability index (Fig. 1g; LiFE: *ϕ* *=* 0.507, CI [0.503, 0.512], ReAl-LiFE: *ϕ* = 0.533, CI [0.528, 0.538]; *P* < 0.001 across all connections). Pruning with SIFT2 yielded reliability indices comparable with those of ReAl-LiFE (Fig. 1g; SIFT2, *ϕ* *=* 0.535, CI [0.530, 0.540]; *P* = 0.812), whereas pruning with COMMIT2 yielded a marginally lower reliability index than ReAl-LiFE (Fig. 1g; *ϕ* = 0.526, CI [0.521, 0.531]; *P* = 0.004).

Next we quantified the improvement in reliability based on the proportion of connections in each quadrant of the (*V*_b_, *V*_w_) plot: a greater proportion in the II quadrant (*V*_b_ > *V*_w_; ‘high-reliability’) as compared to the IV quadrant (*V*_w_ > *V*_b_; ‘low-reliability’) indicates more efficient pruning. Compared to the unpruned connectome, streamline pruning with LiFE yielded fewer connections in the high-reliability (6.8%) than in the low-reliability quadrant (11.1%), although not different from chance (*P* = 0.994, binomial test; Fig. 1e). On the other hand, pruning with ReAl-LiFE yielded significantly more connections in the high-reliability (13.2%) than in the low-reliability quadrant (8.5%; *P* = 0.011; Fig. 1e). Pruning with SIFT2 and COMMIT2 yielded comparable proportions of connections in both the high- and low-reliability quadrants (SIFT2, *P* = 0.399; COMMIT2, *P* = 0.390; Fig. 1e).

Finally, we identified connections exhibiting extreme values of test–retest reliability following ReAl-LiFE pruning (Fig. 1f). The highest test–retest reliability was observed for long-range connections between the frontal and parietal lobes (Supplementary Table 3), which strongly overlap with established white-matter tracts including the superior and inferior longitudinal fasciculus (SLF/ILF), the arcuate fasciculus (AF) and the inferior fronto-occipital fasciculus (IFOF) (Fig. 1h). Conversely, test–retest reliability was least for short-range connections, especially those connecting the middle temporal gyrus with adjacent temporo-occipital regions (Fig. 1i).

**Fig. 2:**
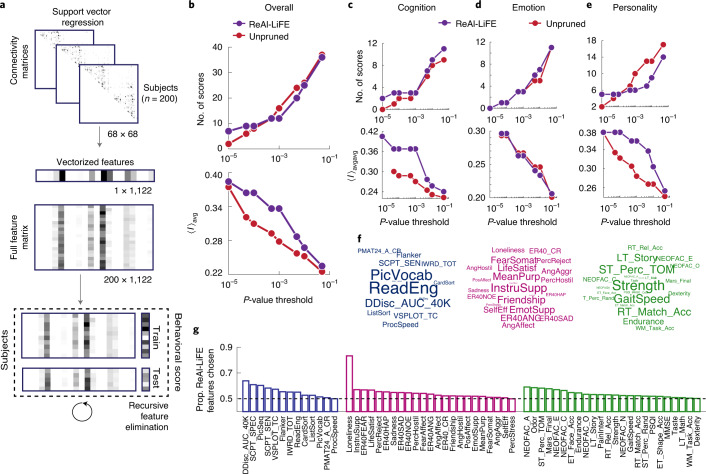
Predicting key cognitive scores using ReAl-LiFE connection weights. **a**, Schematic of the SVR-RFE prediction model. For each participant (*n* = 200), the 68 × 68 whole-brain connectivity matrix was vectorized to give 1,122 connectivity features. Feature vectors from all participants were collated to form a feature matrix of size 200 × 1,122, which was used to predict 60 different behavioral and cognitive scores. Data were divided into training and testing folds, and the prediction model was trained on the train fold using SVR (dashed box). Feature selection was implemented using RFE. **b**, Top: number of scores significantly predicted as a function of the uncorrected *P*-value threshold for predictions based on the number of fibers in the unpruned connectome (red circles) and connection weights in the ReAl-LiFE-pruned connectome (purple circles). Bottom: average correlations between the observed and predicted scores as a function of the uncorrected *P*-value threshold. Other conventions are the same as in the top panel. **c**–**e**, As in **b**, but for scores from the cognition (**c**), emotion (**d**) and personality (**e**) categories. Other conventions are the same as in **b**. **f**, Word clouds showing the different behavioral scores from the cognition (left), emotion (middle) and personality (right) categories, sized based on their prediction accuracy values using ReAl-LiFE connection weights. Larger words indicate better predicted scores. **g**, Proportion of ReAl-LiFE features chosen by the SVR-RFE model for predicting each of the 60 behavioral and cognitive scores using a combined feature set, including both the number of fibers in the unpruned connectome (1,122 features), as well as ReAl-LiFE connection weights (1,122 features). The proportions of features are shown in descending order, separately, for each of the three categories of scores—cognition (blue, *n* = 13), emotion (magenta, *n* = 23) and personality (green, *n* = 24). Source data

As a real-world application of ReAl-LiFE, we asked if streamline pruning with ReAl-LiFE would enable identification of structural connectivity correlates of behavior. For this, we predicted 60 behavioral test scores^13^ spanning three categories—cognition, emotion and personality—of 200 participants (HCP database^9^; Supplementary Data File 1). Behavioral score prediction was performed with a support vector regression (SVR) model using recursive feature elimination (RFE)^14^ (Methods and Fig. 2a). Specifically, we compared predictions made with ReAl-LiFE connection weights as features against those based on the number of fibers in the unpruned connectome.

Across a range of significance thresholds (*α* = 0.00001 to 0.05, uncorrected), the number of behavioral scores predicted significantly by connectome features based on both the number of fibers (Fig. 2b, top, red filled circles) and ReAl-LiFE weights (Fig. 2b, top, purple filled circles) were not different (*P* = 0.906; effect size = 0.011). However, predictions based on ReAl-LiFE weights were more accurate than those based on the number of fibers, as evidenced by the higher average correlations (Fig. 2b, bottom; effect size = 0.660). Similar trends were observed when each category of scores—cognition, emotion and personality—was predicted separately (Fig. 2c–f and Supplementary Fig. 5). Specifically, for cognition and personality scores, ReAL-LiFE weights yielded consistently higher prediction accuracies (average *r* values) than the number of fibers, across the entire range of significance thresholds (Fig. 2c,e, lower panels).

Next we asked which set of connectome features—ReAl-LiFE weights or unpruned connectome fibers—more robustly predicted behavioral scores. For this, we combined both sets of features and quantified the proportion of ReAl-LiFE features selected by the RFE algorithm for the best prediction of each behavioral score. For over 95% (58/60) of score predictions, RFE favored a higher proportion of ReAl-LiFE features, as compared to features from the unpruned connectome (*P* < 0.001, Wilcoxon signed-rank test; Fig. 2g). Additional control analyses for these behavior predictions are reported in the Supplementary Information (Supplementary Fig. 5).

We also analyzed the anatomical features underlying these predictions, focusing on the ‘cognition’ scores. Briefly, reading ability correlated significantly with connectivity in the left frontal cortex, whereas picture vocabulary scores correlated significantly with connectivity within the right parietal cortex (Supplementary Fig. 5 and Supplementary Data File 2).

Our findings indicate that, in addition to yielding more accurate connectomes, ReAl-LiFE connection weights enabled more accurate predictions across a range of behavioral scores, all acquired outside the scanner environment. dMRI-based structural connectivity, quantified following connectome evaluation, may provide a reliable neuroimaging-based biomarker for key cognitive traits.

The ReAl-LiFE algorithm may be developed and improved on several key fronts to overcome its current limitations. First, the current version of the ReAl-LiFE algorithm does not take advantage of parallel computations across multiple GPUs. Moreover, ReAl-LiFE is presently not integrated with multi-CPU acceleration schemes^10^, although our speedups exceed (~8.7×) state-of-the-art numbers reported for these approaches. Combining these CPU-based schemes with our GPU implementation, or implementing parallel computations across multiple GPUs, may yield further speedups of the algorithm.

Second, ReAl-LiFE’s optimization objective, including the sparsity-inducing prior, may be further improved. A key feature of regularized pruning with ReAl-LiFE is the ability to generate connectomes at various, desired levels of sparsity using L1-norm-based regularization, a feature unavailable in the original LiFE algorithm. Yet, such a stringent regularization increases the chances of false negatives (missed fibers)^8,11^. Although we tested for this possibility (Supplementary Fig. 2), other kinds of regularization (for example, L2-norm-based) will need to be systematically evaluated to identify those that minimize false negatives in the connectome. Nonetheless, incorporating this penalty resulted in ReAl-LiFE outperforming LiFE in terms of reducing overfitting, producing more consistent connectomes and increasing test–retest reliability. Although the test–retest reliability analyses were performed with only a limited number of participants (*n* = 5), ReAl-LiFE connections with the highest test–retest reliability mapped onto established white matter tracts^15^. Incorporating additional features and constraints into the objective function—for example, based on brain anatomy, as with COMMIT2^8^—may enable future improvements to the ReAl-LiFE algorithm.

Third, a principled evaluation of the reasons underlying the differences between ReAl-LiFE and other state-of-the-art algorithms—SIFT/SIFT2 and COMMIT2^6–8^—remains to be carried out. ReAl-LiFE pruning times were largely comparable with the other approaches, but demonstrated a marginal advantage for larger connectome sizes. Moreover, ReAl-LiFE differed from the other approaches in the proportion of high-reliability connections retained following pruning. The reasons for these differences need to be investigated carefully. Finally, ReAl-LiFE will need to be directly compared against these competing approaches, in terms of their respective success rates with mapping structural connectivity to behavior. Such a principled comparison is essential for identifying robust structural connectivity bases of higher-order cognitive functions, such as attention, learning and decision-making^15,16^.

More generally, the Subspace Barzilai–Borwein Non-Negative Least-Squares (SBB-NNLS) algorithm, at the heart of ReAl-LiFE, is widely applicable to optimization problems in many real-world applications, including healthcare^17^. Our GPU-accelerated implementation of the SBB-NNLS algorithm has the potential for wide application in diverse domains that go beyond connectome pruning.

## Methods

All experiments were conducted according to protocols approved by the Institute Human Ethics Committee, Indian Institute of Science, Bangalore. Informed written consent was obtained from each participant before the study.

### ReAl-LiFE

#### Description of the LiFE algorithm

The LiFE algorithm models a diffusion signal from the reconstructed whole-brain connectome. LiFE’s optimization algorithm minimizes the error between the modeled and the measured diffusion signal, while eliminating fibers from the connectome that do not contribute to the underlying diffusion signal. The diffusion signal is typically measured along multiple, uniformly sampled gradient directions in space, *N*_*θ*_. For each voxel and gradient direction, this signal is then encoded into a vector ${{{\bf{b}}}}\, \in \,{{R}}^{{{N}_\theta} \times {{N}_{{{\mathrm{v}}}}}}$, where *N*_v_ is the number of voxels in the data. In a given whole-brain connectome, fibers traverse multiple voxels and each voxel may contain many different fibers. The contribution of each fiber f, traversing a voxel v, measured along the gradient direction *θ*, is encoded into a matrix ${{M}}\, \in \,{R}^{{{N}_{{{\mathrm{v}}}}}{{N}_\theta} \times {{N}_{{{\mathrm{f}}}}}}$, where *N*_f_ is the number of fibers in the connectome. The diffusion signal in each voxel is modeled as a weighted sum of the individual fibers traversing the voxel. This can be written as **b** = *M***w**, where ${\bf{w}}\, \in \,{R}^{{N}_{{{\mathrm{f}}}}}$ signifies the contribution (or weight, *w*_f_) of each fiber f to the diffusion signal **b**.

LiFE minimizes the error between the modeled and measured diffusion signal by assigning a non-negative weight to each fiber. This objective is posed as a non-negative least-squares optimization problem:

1 $${\mathop {{\min }}\limits_{{\bf{w}}} \left( {{O}\left( {{\bf{w}}} \right)} \right),\quad {{O}}({{\bf{w}}}) = {\frac{1}{2}}\left\| {{{\bf{b}}} - {{M\bf{w}}}} \right\|^{2},\quad {{\bf{w}}} \ge {0}}$$

Solving this problem imposes substantial memory demands due to the large size of *M*, which is typically 2 GB for a typical connectome with *N*_v_ = 100,000, *N*_f_ = 1 million and *N*_*θ*_ = 64 (refs. ^3,18^). A recent study^18^ overcame this limitation by adopting a more efficient, sparse tensorial representation of *M*. The demeaned diffusion signal ${{M}}_{{{\mathrm{v}}}}\, \in \,{{R}}^{{{{N}}_\theta} \times {{N}}_{{{\mathrm{f}}}}}$ in each voxel v was represented, using sparse Tucker decomposition (STD), as ${{M}}_{{{\mathrm{v}}}} = {{S}}_{0}({{v}}){{D}}{\varPhi _{{{\mathrm{v}}}}}$, where *S*_0_(*v*) is the ‘baseline’ diffusion signal measured in the absence of a diffusion gradient, ${{D}} \in {{R}}^{{{N}}_{\theta} \times {{N}}_{{{\mathrm{a}}}}}$ is a dictionary matrix quantifying the contribution of canonical diffusion ‘atoms’ (*N*_a_ atoms) toward each diffusion direction, and ${{\varPhi }}_{{{\mathrm{v}}}} = {{R}}^{{{N}}_{{{\mathrm{a}}}} \times {{N}}_{{{\mathrm{f}}}}}$ is a sparse, binary matrix whose columns indicate the contribution of each atom to each fiber, in that voxel. Collating *Φ*_v_ for all voxels v into a sparse 3D tensor *Φ*, the modeled (or predicted) diffusion signal may be written as

2 $${{Y}} = {{\varPhi }} \times _{1}{{D}} \times _{2}{{S}}_{0} \times _{3}{{\bf{w}}}^{{{\mathrm{T}}}}$$

where **w** is the vector of all the individual fiber weights and ×_*k*_ represents a matrix product in mode *k*. *M* in equation (1) is now given by ${{M}} = {{\varPhi }} \times _{1}{{D}} \times _{2}{{S}}_{0}$.

With this representation, the optimization problem is solved using an SBB-NNLS algorithm^10^. Briefly, given **w**^0^ as an initial weight vector, the weight updates occur as follows:

$${{{\bf{w}}}}^{({{i}} + 1)} = [{{{\bf{w}}}}^{({{i}})} - {\alpha }^{({{i}})}\nabla {{g}}({{\bf{w}}}^{({{i}})})]^ +$$

where the gradient term is given by

3 $${\nabla }{{g}}({{{\bf{w}}}}) = {{M}}^{{{\mathrm{T}}}}({{M\bf{w}}} - {{\bf{b}}})$$

and *α*^(*i*)^, the step value at each iteration, is given by

$${\alpha }_{\rm{{odd}}}^{({{i}})} = {\frac{{ \langle \nabla {{{\tilde{g}}}}^{({{i}} - 1)},\nabla {{{\tilde{g}}}}^{({{i}} - 1)} \rangle }}{{ \langle {{M}}\nabla {{{\tilde{g}}}}^{({{i}} - 1)},{{M}}\nabla {{{\tilde{g}}}}^{({{i}} - 1)} \rangle }}}$$

for the odd iterations and

$${\alpha }_{{\rm{even}}}^{({{i}})} = {\frac{{ \langle {{M}}\nabla {{{\tilde{g}}}}^{({{i}} - {1})},{{M}}\nabla {{{\tilde{g}}}}^{({{i}} - {1})} \rangle }}{{ \langle {{M}}^{{{\mathrm{T}}}}{{M}}\nabla {{{\tilde{g}}}}^{({{i}} - {1})},{{M}}^{{{\mathrm{T}}}}{{M}}\nabla {{{\tilde{g}}}}^{({{i}} - 1)} \rangle }}}$$

for the even iterations. The tilde denotes the projection of the gradient into the positive space at each iteration and 〈**a**, **b**〉 denotes an inner product of the vectors **a** and **b**.

In its original form, the LiFE algorithm suffered from a few key drawbacks, which needed to be overcome to enable connectome evaluation at scale. First, a CPU implementation of the memory-efficient implementation of the LiFE algorithm still suffered from computational bottlenecks, because every iteration of the algorithm requires a large number (*O*(*N*_v_*N*_*θ*_*N*_f_)) of multiplications, typically, with matrices comprising 10^13^–10^14^ elements. This requirement produced a considerable speed bottleneck when evaluating large connectomes. Second, LiFE (and related pruning algorithms) suffer from another key limitation: there is no explicit provision in LiFE or in other popular pruning algorithms, such as SIFT^7^ or SIFT2^6^, for directly eliminating redundant fibers in the connectome.

#### GPU acceleration

To address the issue of speed, we sought to identify key bottlenecks in LiFE’s algorithm that could be optimized on GPUs. Briefly, the SBB-NNLS optimization algorithm requires several multiplications of the form *Mx* or *M*^T^*y*, where *x* and *y* are generic notations of matrices that are used in various steps of the optimization. We sought to speed up these multiplications with efficient GPU-based computations. The detailed steps are presented as pseudocode in algorithms 1 and 2 in the Supplementary Information. Here we describe these steps briefly.

A key ingredient of our GPU acceleration approach is splitting the computation among voxels, with each CUDA block handling data associated with one voxel. For storage efficiency, the matrix *M* was stored in a sparse tensor (Coordinate list, COO format) with indices into the dictionary matrix *D*, as it was not feasible to use standard sparse matrix multiplication packages. Following STD of *M* (equation (2)), computing *Mx* requires computing linear combinations of columns from *D* while *M*^T^*y* computation requires computation of inner products with columns of *D* (ref. ^18^). The former has a high memory write bandwidth requirement, whereas the latter has a high memory read bandwidth requirement as well as a reduction operation. To address this issue we sorted the *Φ* tensor, stored in the COO format, along the voxel dimension, enabling faster per-voxel execution of both *Mx* and *M*^T^*y* by reducing memory write and read requests, respectively.

We fixed the block size to the warp size of the GPU, which corresponds to the number of threads processing each voxel. Each thread handled one or more diffusion directions, depending on the total number of diffusion directions. Data along the diffusion direction dimension were padded such that its size was a multiple of the warp size, to avoid branching in the kernel code that is to be run on the GPUs. This also permitted maximizing the usage of warp shuffle instructions and reducing shared memory usage. We used shared memory only for storing the final results. In each block, we read up to warp size entries from the sparse tensor in parallel, to leverage memory coalescing advantages, and stored them in thread local memory. The threads in a block then computed on different diffusion directions for the read entries sequentially. We used warp broadcast instructions to share data from thread local memories to all threads in a block. In the case of *M*^T^*y* computation, we used warp shuffle instructions for computing inner products. This freed up resources, potentially allowing more blocks to be scheduled at any given time.

We implemented these algorithms (algorithms 1 and 2 in the Supplementary Information) with the CUDA language for use with NVIDIA GPUs. The results reported in the main text reflect speedups with the CUDA implementation. In addition, we implemented these same algorithms on AMD GPUs with the HIP (Heterogenous Compute Interface for Portability) language, using the HIPIFY package (https://github.com/ROCm-Developer-Tools/HIPIFY). We found that execution times for algorithms 1 and 2 on the NVIDIA GeForce GTX 1080 Ti GPU and the AMD Radeon RX 580 GPU are comparable, and several orders of magnitude faster than their corresponding CPU implementations in Matlab (Supplementary Fig. 1).

#### Regularized evaluation

To address the second issue of redundant fibers, we developed a regularized pruning algorithm, extending that of LiFE^10^. We modified LiFE’s least-squares error minimization objective function to incorporate a regularization term for the weights, such that an L1-norm penalty was incorporated into the objective function: ${{{{O}}}({{{\bf{w}}}}) + \lambda (\left\| {{{\bf{w}}}} \right\|_{1}),\,\,{{{\bf{w}}}} \ge {0}}$, where *λ* is the regularization constant. The gradient calculation in equation (3) now changes to ${{g}}({{{\bf{w}}}}) = {{M}}^{{{\mathrm{T}}}}({{M\bf{w}}}-{{{\mathrm{\bf{b}}}}}) + {\lambda {\bf{1}}}$ where **1** is a vector of all 1s. Similarly, we also implemented L2 regularization for the weights, by adding an L2-norm penalty to the objective function: ${{{O}}({{{\bf{w}}}}) + \lambda \left( {\left\| {{{\bf{w}}}} \right\|_2} \right),{{{\bf{w}}}} \ge {0}}$. The gradient calculation in equation (3) now changes to ${{g}}({{{\bf{w}}}}) = {{M}}^{{{\mathrm{T}}}}({{M\bf{w}}} - {{\bf{b}}}) + {\lambda {{{\bf{w}}}}}$. We tested several values of the penalty *λ*, for both the L1 and L2 regularization (Supplementary Fig. 3).

The estimated fiber weights vector **w** is encouraged to be sparse in LiFE through the non-negativity constraint. Additionally, in ReAl-LiFE, more sparsity is induced, through regularization, on the weights vector.

Although a preliminary version of the method has been published previously^10^, in the present study we advance the ReAl-LiFE algorithm by leveraging the sparsity of **w** to further speed up the GPU implementation of *Mx*. Specifically, whenever a weight vector element *x* is zero, the entire matrix multiplication computation for that step can be skipped, in each thread (step 5 in Supplementary algorithm 1). This yielded a substantial improvement in speedups for the present ReAl-LiFE algorithm (>100–150× for the largest connectome sizes tested) over the version of the algorithm published previously^10^ (~50–100×).

### Diffusion MRI acquisition and preprocessing

#### Dataset *I*

Structural and dMRI scans were acquired on a Siemens Skyra, 3T scanner with a 32-channel head coil, at the HealthCare Global Hospital, Bangalore. A T1-weighted MPRAGE structural scan was acquired before the diffusion scan (1-mm spatial resolution; echo time (TE) = 2.32 ms, repetition time (TR) = 2,300 ms, field of view (FoV) = 240 mm, flip angle = 8°, 256-voxel matrix size, parallel acquisition technique (PAT) with in-plane acceleration factor 2 (GRAPPA)). Diffusion scans were acquired along 64 non-collinear directions with a *b* value of 1,000 s mm^−2^ (2-mm isotropic voxels, TE = 90 ms, TR = 8,900 ms, FoV = 256 mm, 128-voxel matrix size, 68 transversal slices with interleaved slice acquisition; parallel acquisition technique (PAT) with in-plane acceleration factor 2 (GRAPPA), phase encoding direction: A>>P). Two non-diffusion weighted images (*b* = 0 s mm^−2^) were acquired, one at the beginning and one at the end of each scan, respectively. Preprocessing of dMRI images followed previously published protocols^3^. Briefly, the T1 image was first manually aligned to the participant’s anterior commisure–posterior commisure (AC–PC) axis coordinates. Following this, scans were preprocessed to correct for head motion, eddy current-related distortions were corrected using a rigid-body alignment algorithm^3^, followed by alignment to the AC-PC aligned T1 image using the VISTA LAB (Stanford Vision and Imaging Science and Technology) diffusion MRI software package, as part of the Vistasoft suite 2017 (https://github.com/vistalab/vistasoft/). This dataset is available on a Figshare repository^19^.

#### Dataset *S*

We acquired a publicly available, preprocessed dataset as used in the evaluation of the LiFE algorithm^3^. Data were acquired on a General Electric Discover 750 (GE Healthcare), 3T scanner with a 32-channel head coil. Diffusion scans were acquired along 96 non-collinear directions with a *b* value of 2,000 s mm^−2^ (1.5-mm isotropic voxels, TE = 96.8 ms). Ten non-diffusion weighted images (*b* = 0 s mm^−2^) were acquired at the beginning of the scan. Preprocessing steps included correcting distortions arising out of B0 field inhomogeneities as well as participant head motion correction using a rigid-body alignment method^3^. Further details regarding the acquisition and preprocessing steps are available in refs. ^3^ and ^18^.

#### Dataset *H*

We acquired a publicly available dataset from the HCP database^9^. Structural and dMRI scans were acquired on a customized Siemens Connectome Skyra, 3T scanner with a 32-channel head coil. The T1-weighted MPRAGE structural scan was acquired at 0.7-mm spatial resolution, TE = 2.14 ms, TR = 2,400 ms, FoV = 224 × 224 mm^2^, flip angle = 8°, PAT with in-plane acceleration factor 2. Diffusion scans were acquired along 270 non-collinear directions using multi-shell imaging with *b* values of 1,000, 2,000 and 3,000 s mm^−2^ (1.25 mm isotropic voxels, TE = 89.5 ms, TR = 5,520 ms, FoV = 210 × 180 mm^2^, 164 × 144 matrix size, multiband-factor = 3, phase encoding direction: L>>R and R>>L). A total of 18 non-diffusion weighted images (*b* = 0 s mm^−2^) were acquired, interspersed throughout the scan. Preprocessing steps included B0 intensity normalization, susceptibility-induced distortion correction, eddy current and participant head motion correction, and gradient nonlinearity correction. Finally, the diffusion images were registered to the structural (T1-weighted) image. For all our analyses, we used HCP’s minimally preprocessed data^9^.

#### Dataset *M*

This dataset was provided as part of an international Tractography Challenge^5^. Briefly, the authors used one dataset from the HCP database^9^ to manually delineate 25 known fiber bundles and their corresponding termini regions (regions of interest, or ROIs) in the human brain^5^; these were termed ‘ground-truth’ bundles. Next, using these ground-truth bundles, diffusion MRI data were simulated corresponding to a *b* value of 1,000 s mm^−2^ and 32 gradient directions using the Fiberfox software. A T1-weighted image was also simulated. The authors provide two datasets—one artifact-free dataset with no noise and one dataset with added artifacts such as head motion, susceptibility-induced distortion, eddy currents, spiking noise, ghosting and ringing artifacts, as well as Gaussian noise.

In addition to the dataset already provided by the authors, we simulated a second, independent dataset with the same parameters, using Fiberfox^5^ version 2018.09.99. Briefly, we generated one diffusion MRI dataset with *b* = 1,000 s mm^−2^ and 32 gradient directions (2-mm isotropic voxels) in the A>>P (anterior to posterior) phase encoding direction. For each dataset, we used a four-compartment model to simulate the (1) inter-axonal, (2) intra-axonal, (3) gray matter (GM) and (4) cerebrospinal fluid (CSF) tissue response profiles. Parameter values corresponding to each compartment model are listed in Supplementary Table 1. All other parameters were set to their respective, default values. We also simulated an additional non-diffusion weighted image (*b* = 0 s mm^−2^) with the phase encoding direction reversed (P>>A). Individual masks used for each compartment as well as the anatomical T1 image were the same as those provided in the original dataset. Preprocessing the simulated data involved denoising with MRtrix3^20^ followed by motion correction and susceptibility-induced distortion correction^20^. Finally, the diffusion MRI scan was aligned to the T1 image.

#### HCP datasets for studying structure–behavior relationships

For predicting behavioral scores using structural connectivity, we used minimally preprocessed diffusion MRI datasets from the HCP database^9^. We utilized data from *n* = 200 participants with an equal number of males and females (*n* = 100 each) for whom both dMRI data as well as behavior data was available (Supplementary Table 2; participant IDs). These participants included 60 participants who were matched for age, gender and handedness from a previous study^21^. The remaining 140 participants were drawn in chronological order from the HCP database to ensure gender and age parity (*n* = 100 females: mean = 29.2 years, s.d. = 3.7 years; *n* = 100 males: mean = 28.5 years, s.d. = 3.9 years). We confirmed that there was no significant difference between the average age of male and female participants (*P* = 0.223, two-sample *t*-test).

### Tractography and generating whole-brain connectomes from dMRI data

#### Datasets I, S, H and M

For each of these datasets, we used a standard tractography pipeline available with MRtrix3^20^. This pipeline comprises the following steps. We first performed a five-tissue-type segmentation on the T1 image to separate out the (1) cortical GM, (2) subcortical GM, (3) white matter, (4) CSF and (5) other pathological tissue. Next, a constrained spherical deconvolution (CSD) algorithm was employed to estimate the fiber orientation distribution (FOD) in each voxel^20^, with the maximum harmonic order at 8 (default value). Finally, anatomically constrained probabilistic tractography was performed using dynamic seeding^7^. The maximum fiber length cutoff and FOD amplitude threshold for datasets *I*, *S* and *M* were set to default values (200 mm and 0.1, respectively). For dataset *H*, the maximum length cutoff and FOD amplitude threshold were set to 250 mm and 0.06, respectively. Following this, we constructed whole-brain connectomes with specific fiber counts, as indicated in the respective sections in the main text. For the behavioral score predictions with 200 participants’ dMRI data from the HCP database, and for the test–retest reliability analyses with five participants’ data from the HCP Retest database, dMRI preprocessing and connectome generation followed the same protocol as indicated for dataset *H*.

#### Ensemble tractography (dataset *S*(ET))

Tractography algorithms include multiple parameter settings with many degrees of freedom. Ensemble tractography seeks to overcome biases associated with specific parameter choices by estimating several connectomes, one for each choice of parameter value, and subsequently combining them into an ‘ensemble’ connectome^10^. For ensemble tractography, we used dataset *S* to generate five whole-brain connectomes, with the maximum radius of curvature of fibers set to one of five parameter values (0.25 mm, 0.5 mm, 1 mm, 2 mm and 4 mm). Each connectome was generated by seeding fibers at the GM–white matter interface. Next, we combined these individual connectomes to form the ensemble connectome. We generated two connectomes: a smaller, 0.8-million-fiber connectome for streamline pruning with LiFE and a larger 1.6-million-fiber connectome for pruning with ReAl-LiFE (for details see the section Estimating the regularization parameter *λ*).

### Quantifying ReAl-LiFE performance as speedups and fits to data

#### Quantifying speedups

For each of the datasets *I, S* and *H* and for every connectome size (Fig. 1c), we first pruned streamlines with the original LiFE optimization algorithm^3^ for 500 iterations. Next, we pruned streamlines with the same connectome using the GPU-accelerated version of LiFE (with no regularization), again for 500 iterations. Speedup of the GPU-accelerated pruning as compared to CPU pruning was computed as

$${{{\mathrm{Speedup}}}} = \frac{{{{t}}({{{\mathrm{LiFE}}}}_{{{{\mathrm{CPU}}}}})}}{{{{t}}({{{\mathrm{LiFE}}}}_{{{{\mathrm{GPU}}}}}) + {{t}}({{{\mathrm{Overhead}}}}_{{{{\mathrm{GPU}}}}})}}$$

where *t*(LiFE_CPU_) is the time taken for 500 iterations of the LiFE algorithm on the CPU, *t*(LiFE_GPU_) is the time taken for 500 iterations of the GPU-accelerated LiFE and *t*(Overhead_GPU_) is the GPU overhead time corresponding to the time taken for data transfer between the CPU and GPU memory and other preprocessing steps, such as sorting along the voxels dimension.

To calculate the speedup scaling factor, we fit a function of the form ${{y}} = {{a}} + {{b}}\,{{{\mathrm{log}}}}({{x}})$ to the speedups, where *y* is the speedup, *x* is the parameter (*N*_v_, *N*_*θ*_, *N*_f_), *a* is the intercept and *b* is the slope. We quantified the scaling factor (*q*) as change in speedup from the lowest to the highest value for each of the parameters *N*_v_, *N*_*θ*_ and *N*_f_.

We compared the speedups of GPU-accelerated LiFE over a second state-of-the-art algorithm, SIFT^7^. We tested for speedups for each of the three datasets—*I*, *H* and *S*—on a connectome of one million fibers (*N*_f_ = 10^6^), using the respective default values for parameters *N*_v_ and *N*_*θ*_. To ensure a fair comparison, we first pruned streamlines from each unpruned connectome with the GPU-accelerated LiFE, systematically running the optimization algorithm from 50 to 500 iterations (steps of 50 iterations). Next, we pruned the same (respective) unpruned connectome with SIFT, albeit to the exact same size as the corresponding LiFE-pruned connectome. In other words, the termination criterion for SIFT was specified to match the number of fibers in the LiFE-pruned connectome. We then defined the convergence time *t*_c_ as the iteration at which the change in the objective function *O*(*t*) over the last ten iterations was less than 0.1% of its initial value ${{{O}}({{t}}_{{{\mathrm{c}}}} + 10) - {{O}}({{t}}_{{{\mathrm{c}}}}) < {{\varDelta }} \cdot {{O}}({{{t}}_{0}})}$, where *O*(*t*_0_) is the initial value of the objective function and *Δ* = 0.001. For each dataset, we then computed the speedup of GPU-accelerated LiFE over SIFT at convergence as the average of the speedups across the nearest two multiples of 50 iterations. Speedups were compared based on total execution times of each algorithm from start to finish, including all overheads associated with loading data into memory.

We also compared the speedups of GPU-accelerated LiFE over yet another a state-of-the-art algorithm, COMMIT2, which incorporates anatomically informed priors into its objective^8^. As before, for each of the three datasets (*I*, *H* and *S*), we computed speedups of a connectome of one million fibers (*N*_f_ = 10^6^), with default parameter values for *N*_v_ and *N*_*θ*_. To enable a fair comparison, in this case, we first pruned the connectome with COMMIT2 until convergence, with all default parameters. Next, we pruned the same (respective) unpruned connectome with GPU-accelerated LiFE to within 1% of the initial size of the corresponding COMMIT2-pruned connectome. In this case, we pruned the ReAl-LiFE streamlines to the size of the COMMIT2-pruned connectome, because the converse approach—pruning the COMMIT2 streamlines to match the ReAl-LiFE-pruned connectome, at convergence—required over 500 iterations of the COMMIT2 algorithm. As with SIFT, speedups were compared based on total execution times of each algorithm from start to finish, including all overheads associated with loading data into memory.

Finally, we tested a dockerized version of the LiFE algorithm (10.25663/bl.app.104) to optimize a one-million fiber connectome for each of the three datasets (500 iterations). We found that the run times were comparable to those of CPU-LiFE, as reported in Supplementary Fig. 1 (dataset *H*: CPU-LiFE 17.9 h, dockerized LiFE, 23.1 h; dataset *I*: CPU-LiFE 3.0 h, dockerized LiFE 3.3 h; dataset *S*: CPU-LiFE 8.2 h, dockerized LiFE 8.4 h).

#### Quantifying duplication of fiber weights

We tested ReAl-LiFE’s ability to prune away duplicate (redundant) fibers and compared its performance with the LiFE and SIFT2 algorithms^6^. For this we used two approaches. In the first approach, we used dataset *M* to generate a whole-brain connectome comprising 0.5 million fibers. Next, we used this connectome to simulate a noise-free diffusion signal^5^. We then created two, randomly ‘jittered’, near-identical versions of this connectome (C1 and C2), by randomly perturbing the spatial coordinates of 10% of the nodes in each fiber, randomly by ±0.01%. We combined these perturbed connectomes to create a single connectome comprising one million fibers. Finally, we pruned streamlines in the combined connectome with LiFE and ReAl-LiFE, using the noise-free diffusion dataset simulated in the previous step. For pruning with SIFT2, we followed a slightly different approach. SIFT2, unlike LiFE or ReAl-LiFE, does not prune out (eliminate) any fiber. To facilitate a fair comparison with LiFE and ReAl-LiFE, we first pruned the combined connectome with SIFT^7^ followed by pruning with SIFT2 to obtain fiber weights of the unpruned fibers retained by SIFT. We sought to perturb the fibers across the two connectomes before pooling them, rather than combining two connectomes with identical, duplicated fibers; in the latter case, each algorithm yielded identical weights across each pair of duplicate fibers because none of the algorithms could break ties across identical fibers.

Following pruning, we computed a ‘uniqueness’ index quantifying the normalized difference in weights between copies of corresponding fibers as

$${{{\mathrm{\zeta }}}}_{{{{\mathrm{uniq}}}}} = \frac{{|{{w}}_{1} - {{w}}_{2}|}}{{({{w}}_{1} + {{w}}_{2})}}$$

A higher value of ζ_uniq_ indicates a higher tendency to prune out redundant fibers and to retain only one copy of the two near-identical fibers.

In the second approach, we created a ‘trimmed’ version of the whole-brain connectome from dataset *M* (same as estimated in the previous approach) by trimming out 5% of the nodes from each terminus of each fiber. We then combined the original whole-brain connectome with this jittered copy, followed by pruning with LiFE, ReAl-LiFE and SIFT2, as before.

#### Estimating the regularization parameter *λ*

We sought to compare the performance of LiFE with ReAl-LiFE in terms of model fit. Because the L1-regularization penalty in ReAl-LiFE yields systematically sparser connectomes as compared to LiFE, to enable fair comparisons of the model fit we matched the summed weights (L1-norm) of the fibers, following pruning with each approach (Supplementary Fig. 3). To permit this, we generated a larger initial connectome, with 2× the number of fibers for pruning with ReAl-LiFE, as compared to LiFE, and sampled *λ*, in the range of [10^−8^, 1] in logarithmically spaced steps (Supplementary Fig. 3). For datasets *I* and *M*, we compared the cross-validated root-mean-square error (r.m.s.e.) by generating a connectome with one million fibers, followed by pruning with LiFE (unregularized), against the accuracy of a connectome generated with two million fibers, followed by pruning with ReAl-LiFE. For dataset *S*(ET), we generated two ensemble connectomes: one with 0.8 million fibers (for pruning with LiFE) and another with 1.6 million fibers (for pruning with ReAl-LiFE). These ensemble connectomes were assembled from five smaller whole-brain connectomes generated with 160,000 and 320,000 fibers respectively. We then chose the *λ* that matched the L1-norm of weights across both pruning approaches. For datasets *I*, *M* and *S*(ET), *λ* values of 0.006, 0.01 and 0.01, respectively, provided this match (Supplementary Fig. 3); unless otherwise specified, the same configurations and regularization parameter values were used for all subsequent analyses (for example, Fig. 2 and Supplementary Figs. 2–4).

#### Quantifying the model fit

For each dataset (datasets *I*, *S*(ET) and *M*), we evaluated the performance of the pruning algorithm in two ways—by testing for overfitting and consistency. To test for overfitting, we first generated a whole-brain connectome (C1) with one diffusion dataset (D1; Supplementary Fig. 3). Next, we pruned C1 with LiFE and ReAl-LiFE using dataset D1 as ground truth to obtain a predicted diffusion signal (P1; Supplementary Fig. 3). Finally, we computed the voxel-wise, cross-validated r.m.s.e. between the predicted diffusion signal P1 and a second, independently acquired diffusion dataset (D2; Supplementary Fig. 3) from the same participant. For dataset *M*, D2 was independently simulated from the same underlying ground-truth connectome (see section Diffusion MRI acquisition and preprocessing). We then computed the distribution of voxel-wise r.m.s.e.s, following pruning with LiFE and ReAl-LiFE, and also computed their pairwise differences (ReAl-LiFE – LiFE; Supplementary Fig. 3).

To test for consistency (Supplementary Fig. 4), we generated two whole-brain connectomes (C1 and C2) from two independently acquired diffusion datasets (D1 and D2, respectively), both from the same participant (Supplementary Fig. 4). Next, we pruned C1 (with LiFE or ReAl-LiFE) with dataset D2 as ground truth and, similarly, pruned C2 with dataset D1 as ground truth (Supplementary Fig. 4). We then chose the fibers assigned the top 50th percentile of the weights, to predict the diffusion signals P1 and P2 (Supplementary Fig. 4). Finally, we computed the voxel-wise, r.m.s.e. between the predicted diffusion signals P1 and P2 (Supplementary Fig. 4). As before, we computed the distribution of voxel-wise r.m.s.e.s, following pruning with LiFE and ReAl-LiFE, and also computed their pairwise differences (ReAl-LiFE – LiFE; Supplementary Fig. 4).

### Test–retest reliability analysis, HCP retest data

#### Estimating structural connectivity features

For each participant in the HCP retest dataset (*n* = 5; blue indices in Supplementary Table 2), we estimated two whole-brain connectomes, one comprising one million fibers and a second two million fibers (see ‘Tractography and generating whole-brain connectomes from dMRI data’). This was done both for the original dataset (dataset 1) and the retest dataset (dataset 2). For each visit’s data, we employed FreeSurfer’s anatomical parcellation based on the Desikan–Killiany atlas comprising 34 regions on each hemisphere to construct two 34 × 34 structural connectivity matrices (one per hemisphere)^21^. We computed five kinds of structural connectivity matrix: (1) unpruned, (2) ReAl-LiFE pruned (*λ* = 0.01, two-million-fiber connectome), (3) LiFE pruned (one-million-fiber connectome), (4) SIFT2 pruned (one-million-fiber connectome) and (5) COMMIT2 pruned (one-million-fiber connectome). For each connectivity matrix, the (*i*, *j*)th entry indicates the number of fibers, or the sum of pruned fiber weights (after pruning with either ReAl-LiFE, LiFE, SIFT2 or COMMIT2), across all fibers connecting regions *i* and *j*, respectively. Because diffusion MRI does not provide information regarding the direction of connectivity, each matrix was symmetric about the diagonal. For these analyses we considered only connections between pairs of intra-hemispheric regions and ignored diagonal elements of the connectivity matrix, that is, connections that originate and terminate within the same ROI. As a result, the total number of connectivity features across both hemispheres was 1,122 (^34^C_2_ connections per hemisphere × 2 hemispheres). To limit noisy estimates of test–retest reliability metrics, we chose only connections with the top 50th percentile of fibers (561 connections), for the test–retest reliability analysis.

#### Within-participant variability (*V*_w_)

To compute within-participant variability, for each participant, and for each connection in the connectivity matrix, we computed a normalized difference index between the connectivity metrics of the first dataset and the second dataset, that is

$${V_{{\rm{w}}_k}^i} = {\frac{{\left| {{c_1^i} - {c_2^i}} \right|_{k}}}{{({c_1^i} + {c_2^i})_{k}}}}$$

where *k* denotes the participant, *i* denotes the connection, *i* = 1, 2, ..., 561, and *c*_1_ and *c*_2_ denote the connectivity strengths based on datasets 1 and 2 (two visits), respectively. We then computed average within-participant variability for each connection as

$${V_{\rm{w}}^i} = {\left\langle {V_{{\rm{w}}_k}^i} \right\rangle _{k}}$$

where the angle brackets ${\left\langle \cdot \right\rangle _k}$ denote the average across the participants.

#### Between-participant variability (*V*_b_)

To compute between-participant variability, for each connection in the connectivity matrix, we first computed a normalized difference index between the connectivity metrics of every participant’s dataset *m* and every other participant’s dataset *l* (*m* ≠ *l*), that is

$${V_{{\rm{b}}_{lm}}^i} = {\frac{{\left| {{c_{1l}^i} - {c_{2m}^i}} \right|_{lm}}}{{({c_{1l}^i} + {c_{2m}^i})_{lm}}}}$$

where *i* denotes the connection, *i* = 1, 2, ..., 561, and *c*_1_ and *c*_2_ denote the connectivity strengths based on datasets 1 and 2. We then computed average between-participant variability as

$${V_{\rm{b}}^i} = {\left\langle {V_{{\rm{b}}_{lm}}^i} \right\rangle _{l,\,m}},\,{l \ne m}$$

where the angle brackets ${\left\langle \cdot \right\rangle _{l,\,m}}$ denote the average across each pair of participants.

Finally, for each connection *i*, we computed a difference in variability between pruned and unpruned connectivity as

$${\Delta V_{\rm{w}}} = {V_{\rm{w}}^{\rm{pruned}} - {V_{\rm{w}}^{\rm{unpruned}}}}\,{{{\mathrm{and}}}}\,{\Delta V_{\rm{b}}} = {V_{\rm{b}}^{\rm{pruned}} - {V_{\rm{b}}^{\rm{unpruned}}}}$$

#### Reliability

Finally, for each connection *i*, we computed a reliability metric *ϕ* as

$${\phi _i} = {\frac{{V_{\rm{b}}^i}}{{V_{\rm{b}}^i + V_{\rm{w}}^i}}}$$

That is, the reliability of connection *i* was taken as the ratio of the between-participant variability to the total variability, including between- and within-participant components, following ref. ^12^. Using this reliability metric computed using the HCP retest data, we identified connections with the highest and lowest reliability metrics.

### Analysis of structure–behavior relationships

We tested whether structural connectivity could explain inter-individual variations in various cognitive scores, with data from *n* = 200 participants from the HCP database. Specifically, we performed SVR-based prediction of cognitive scores with structural connectivity features estimated either from the unpruned connectome (number of fibers) or by pruning streamlines with ReAl-LiFE or SIFT (connection weights).

#### Behavioral scores

For each of the *n* = 200 participants drawn from the HCP database, we predicted 60 behavioral scores (Supplementary Data 1) from dMRI connectivity^9^. These 60 scores were chosen based on selection criteria employed in previous studies that sought to predict these scores from functional MRI connectivity^13^. In addition to selecting the 58 scores employed in these studies, we included the in-scanner score related to performance accuracy in the shape-matching subtask of the emotion task (Emotion_Task_Shape_Acc, Supplementary Data 1) and replaced the overall accuracy in the relational task with accuracies in each of its subtasks (object matching/Relational_Task_Match_Acc and object relation/Relational_Task_Rel_Acc). Both state and trait scores were included, and age-adjusted scores were used, wherever available. Importantly, behavioral score selection was agnostic to the results of our prediction analyses. Broadly, these behavioral scores fell into three major categories: cognition (*n* = 13), emotion (*n* = 23) or personality (*n* = 24)^22^. These scores also included nine behavioral performance scores recorded during the task-functional MRI sessions. These included accuracy scores in all subtasks of each task, except for the working-memory task, for which we only chose the overall performance accuracy across all subtasks. Further details are provided in Supplementary Data 1.

#### Estimating structural connectivity features

For each participant in the HCP dataset (*n* = 200), we estimated a whole-brain connectome comprising one million fibers (see ‘Tractography and generating whole-brain connectomes from dMRI data’). As with the test–retest reliability analysis, we used the FreeSurfer anatomical parcellation based on the Desikan–Killiany atlas comprising 34 regions on each hemisphere to construct a 68 × 68 structural connectivity matrix^21^. We computed two kinds of structural connectivity matrix—(1) unpruned and (2) ReAl-LiFE-pruned (*λ* = 0.01)—where the (*i*, *j*)th entry in each matrix indicates the number of fibers, or the sum of ReAl-LiFE-pruned fiber weights, for fibers connecting regions *i* and *j*, respectively. We also carried out predictions with a combination of features (1) and (2) (Fig. 2g). Because diffusion MRI does not provide information regarding the direction of connectivity, each matrix was symmetric about the diagonal. In addition, for these analyses we considered only connections between pairs of intra-hemispheric regions and also set all diagonal elements of the connectivity matrix (connections that originate and terminate within the same ROI) to zero. As a result, the total number of connectivity features across both hemispheres was 1,122 (^34^C_2_ connections per hemisphere × 2 hemispheres).

#### Prediction model

For predicting behavioral scores using structural connectivity as features, we used an SVM-based regression model with a linear kernel (‘fitrsvm’ function in MATLAB), along with RFE^14^. Behavioral scores were standardized by *z*-scoring, prior to model fitting. The 1,122 connection features were organized into a feature matrix having dimensions 200 × 1,122, where each row corresponds to a participant and each column corresponds to one of the 1,122 connections (features). We employed RFE to identify features with the largest weight magnitudes in the linear prediction model that provided the highest generalized cross-validation accuracy. We describe the algorithm briefly below (a more detailed description is provided in ref. ^14^).

In the first stage of the RFE, the data were divided into *N* folds. During each iteration *i*, *N* − 1 of these were used for training and the *N*th fold was reserved for testing. In the second stage, for each training set (*N* − 1 folds), the training data were further divided into *K* folds. Subsequently, each of these *K* folds was left out exactly once and the SVR model was trained on the *K* − 1 folds. In each of the *K* iterations, the estimated regression coefficients were used to predict the behavioral scores of the left-out fold (*N*th fold) from the first stage, and a correlation between the predicted scores and the observed scores of the test fold was computed. At the end of the second stage (*K* iterations), the *β* weights (regression coefficients) and correlation values were averaged across the *K* iterations to obtain robust estimates. Next, based on these average *β* weights, the bottom 10% of the features were discarded. This marks the end of the second stage. The second stage of the RFE was repeated until all features were eliminated. Finally, we chose the set of features, say *F*_*i*_, that yielded the maximum correlation between the observed and predicted scores.

The above procedure was repeated *N* times, leaving one fold for testing each time. At the end of the *N* iterations, we averaged (across the *N* folds), the final set of estimated *β* weights (<*F*_*i*_>_*i*_). We repeated the entire RFE procedure (first and second stage) for 100 runs, to robustly estimate the top features and predict behavioral scores. For these analyses, we chose *N* = 10 and *K* = 5.

#### Comparing predictions based on the number of fibers and ReAl-LiFE connection weights

We compared the predictions based on ReAl-LiFE weights with those based on the number of fibers. We computed the number of significant predictions (based on the correlation between the observed and predicted scores) for each feature set (ReAl-LiFE weights and number of fibers) for a range of different levels of significance *α*, ranging from *α* = 0.00001 to *α* = 0.05. For each value of *α* we computed the number of scores for which the *P* value for the correlation between the observed and predicted score was less than *α*. We also computed average correlation coefficient values across all significant scores at each *α* level. The number of significant scores and the average correlation coefficients, at each *α* level, for predictions based on each set of features, and for the different score categories, are plotted in Fig. 2.

#### Univariate correlations between connectivity and behavioral scores

To understand the anatomical relevance of the ReAl-LiFE connections predicting behavioral scores, we chose the five best predicted scores in the ‘cognition’ category. For each score, we identified the top connections that contributed most to the prediction, based on the *β* weight assigned to the connection by the SVR-RFE prediction model. For each such connection, we then computed three additional connectivity metrics: (1) the number of fibers post pruning with ReAl-LiFE, (2) streamline volume and (3) streamline length (voxels intersected). For each connection, we then computed univariate correlations between each of these connectivity metrics and the corresponding behavioral score. Univariate correlations (*r* and *P* values) are reported based on robust correlations. This procedure allowed us to identify connectivity metrics, strongly predictive of behavioral scores, that were most strongly correlated with the respective behavioral score.

#### Control analyses

We performed three control analyses for behavioral score predictions, with ReAl-LiFE connection weights as features.

##### Predicting five minimally correlated scores

To account for correlations among the 60 behavioral scores, we selected a subset of five minimally correlated scores, following the same procedure as in ref. ^13^. We recapitulate their approach as follows. First, a pair of scores with an absolute correlation of less than 0.1 was chosen at random. Subsequently, three behavioral scores were selected, one at a time, so that each new score correlated minimally with the existing set of scores (|*r*| < 0.01). This procedure was repeated 100 times, resulting in 100 such sets of five minimally correlated scores. Finally, the subset of five scores with the least maximum absolute mutual correlation was selected. These scores corresponded to personality extroversion (HCP field: NEOFAC_E), emotion recognition (ER40HAP and ER40NOE), picture vocabulary (PicVocab) and processing speed (ProcSpeed); these five scores are not identical to those in ref. ^13^, possibly because, unlike their study, we employed age-adjusted scores, wherever these were available. In our analyses, these scores exhibited a non-significant maximum absolute correlation of *r* = 0.059 (*P* = 0.403).

##### Controling for head motion confounds

To account for confounding of head motion on behavioral score predictions, we repeated predictions with the SVR-RFE model after regressing out the effects of motion parameters from the behavioral scores, again following a procedure closely similar to that of ref. ^13^. From each participant’s dMRI scan, six head-motion-related parameters were extracted: three parameters corresponding to rotations (about the *x*, *y* and *z* axes, respectively) and three parameters corresponding to translations (along the *x*, *y* and *z* axes, respectively). We fit a multiple linear regression model with the motion parameters as the independent variables to fit each behavioral score. Subsequently, the residual corresponding to each prediction was fit with the SVR-RFE model with ReAl-LiFE weights as features. If the residual could not be fit well, this would indicate that the inter-participant behavioral score variations could not be explained by structural connection features over and above what could be explained with the motion parameters alone.

##### Permutation test for RFE predictions

Finally, we corrected for a potential bias of the RFE algorithm toward positive prediction accuracies (*r* values). Because, at each iteration, the RFE algorithm selects features with the highest numerical value of the generalized cross-validation accuracy, average prediction accuracies across iterations could be positively biased. To control for this bias, we performed a random permutation test in which we shuffled participant labels 100 times across each behavioral score and structural connectivity feature and generated a null distribution of prediction accuracies (*r* values). Because generating the null distribution for each behavioral score is computationally expensive, each *r* value in the null distribution (100 permutations) was computed by averaging across, at most, ten iterations of the RFE algorithm. The *P* value was computed as the proportion of observations in the null distribution that were greater than the actual prediction accuracy (actual *r* value; Supplementary Fig. 5).

#### Statistical tests

Unless otherwise stated, all pairwise statistical comparisons were performed with the nonparametric Wilcoxon signed-rank test (for example, Fig. 1 and Supplementary Fig. 2). Voxel-wise r.m.s.e.s post pruning with LiFE and ReAl-LiFE (Supplementary Figs. 3 and 4) were compared using a Kolmogorov–Smirnov test. All correlations were computed based on ‘robust’ correlations: we report values of the ‘bend correlation’ (Fig. 2b–f,h and Supplementary Fig. 5), an approach that accounts for univariate outliers in the data^23^. Unless otherwise specified, multiple comparisons corrections were carried out with the Benjamini–Hochberg approach, at a significance level of *P* = 0.05. To compare the execution times of the GPU implementation of LiFE with those of the CPU implementation of LiFE as a function of the number of fibers *N*_f_ (Supplementary Fig. 1), we employed a two-way analysis of variance with the implementation (CPU/GPU) and the number of fibers as factors. Effect sizes were quantified as Cohen’s *d*. Statistical significance values are reported as ***P < 0.001, **P < 0.01 and *P < 0.05.

### Hardware and software specifications

All analyses described in this study were performed on a desktop computer with the following hardware and software specifications:

- CPU: eight cores; Intel(R) Xeon(R) CPU E5-2623 v3 @ 3.00 GHz
- GPU: one NVIDIA GeForce GTX 1080 Ti (or) AMD Radeon RX 580
- RAM: DDR4, 4 × 16 GB 1,866 MHz (total 64 GB)
- Hard disk space: 447 GB (64 GB configured as swap)
- Operating system: Ubuntu 16.04
- Software: MATLAB R2017b (64 bit); CUDA toolkit 9.0.

We performed all our benchmarking experiments with only one CPU core and one GPU. GPU code binaries were compiled using the ‘nvcc’ compiler with the ‘-ptx’ flag. Because the original LiFE package was implemented in Matlab, integrating the CUDA or HIP implementation with the ReAl-LiFE package requires Matlab support for GPU computation. Matlab support is available for NVIDIA GPUs, but is currently unavailable for AMD GPUs (https://www.mathworks.com/help/parallel-computing/gpu-support-by-release.html). The end-to-end version of the code, integrated with ReAl-LiFE, is currently available for NVIDIA GPUs (Code availability section), and will be released for AMD GPUs as soon as Matlab support for the latter hardware becomes available.

### Reporting Summary

Further information on research design is available in the Nature Research Reporting Summary linked to this Article.

### Supplementary information

Supplementary Information Supplementary Results, Algorithms 1 and 2, Tables 1–3, Figs. 1–5 and References.

Reporting Summary

Supplementary Data 1 List of cognitive scores used for behavioral prediction.

Supplementary Data 2 Correlations between the best predicted cognitive scores fiber features for the top connections.

### Source data

Source Data Fig. 1 Data to reproduce Fig. 1.

Source Data Fig. 2 Data to reproduce Fig. 2.

## Data Availability

Dataset *I* has been deposited into a figshare repository^19^. Dataset *S* is a part of the original LiFE algorithm and can be accessed from the associated repository^18^. Dataset *H* and all HCP data can be accessed from the HCP database^9^. Dataset *M* is available on the Zenodo repository^5^. Source data are provided with this paper.

## Code availability

The custom code for reproducing all figures is available on figshare^19^. The current version of the ReAl-LiFE algorithm is available on Code Ocean^24^. In addition, to streamline pruning, the code pipeline integrates with a standard atlas (HCP-MMP atlas^9^) to label brain regions connecting each pair of streamlines. The pipeline also integrates with the TractSeg tool^25^ for automated tract labeling.
