# Supplementary material for: GPU-accelerated connectome discovery at scale
Source: Nat Comput Sci. 2022 May 30;2(5):298–306. doi: 10.1038/s43588-022-00250-z (PMC10766542; doi:10.1038/s43588-022-00250-z)
Supplement: Supplementary file 1 — Supplementary Results, Algorithms 1 and 2, Tables 1–3, Figs. 1–5 and References. [file 43588_2022_250_MOESM1_ESM.pdf]

nature portfolio

https://doi.org/10.1038/s43588-022-00250-z

Supplementary information

GPU-accelerated connectome discovery at scale

In the format provided by the authors and unedited

Supplementary Information: Results

Speed bottlenecks with standard connectome evaluation tools

Linear Fascicle Evaluation (LiFE) is a state-of-the-art connectome evaluation technique that enables pruning out spurious fibers from oversampled connectomes. LiFE's streamline pruning algorithm models (predicts) the diffusion signal from an estimated whole-brain connectome and eliminates fibers successively until the cross-validation error between the modeled and measured diffusion signal is minimized. The dMRI diffusion signal is measured along a number of gradient directions (N_θ), and encoded in a vector of size $\mathrm{N}_\theta\mathrm{N}_\mathrm{v}$ where N_v is the number of voxels in the diffusion MRI scan (b_m; Figure 1a). Typically, each fiber in the connectome traverses multiple voxels and each voxel is traversed by many fibers. The contribution of each fiber f, traversing voxel v, to the diffusion signal along gradient direction θ is encoded in a matrix M of size $\mathrm{N}_\mathrm{v}\mathrm{N}_\theta \times \mathrm{N}_\mathrm{f}$, where N_f is the number of fibers in the connectome. Finally, the diffusion signal in each voxel is modeled as a weighted sum of the contributions from all fibers passing through it (Figure 1a): $\mathrm{b}_p = \mathrm{Mw}$, where w, is the "fiber weight" vector of size N_f , with non-negative entries representing the contribution (or weight, w_f) of each fiber f to the modeled or predicted diffusion signal (b_p). The fiber weight vector, w, is estimated with optimization approaches (e.g., SBB-NNLS, Methods) that seek to minimize the disparity between the modeled (b_p) and measured diffusion signal (b_m), the latter being acquired, typically, in a second dMRI scan. This optimization is the major time-consuming step in the LiFE algorithm and is implemented as a series of array multiplications in two key evaluation functions (Supplementary Algorithms 1 and 2; Methods).

We tested the recent version of the LiFE algorithm[1] with a desktop CPU (Intel(R) Xeon(R) CPU E5-2623 v3, 3.00GHz processor) on a 1-million fiber connectome from the Human Connectome Project (HCP)[2] database (Methods). The LiFE algorithm required about 17.9 hours to converge (>500 iterations). Execution times increased monotonically with

connectome size, with the smallest connectome (N_f=50,000) requiring 1.5 hours, compared to 36.7 hours for the largest connectome (N_f=2,000,000) (Supplementary Figure 1a, left, yellow) Similarly, evaluation times scaled systematically with both the spatial resolution (number of voxels) in the dMRI scan (Supplementary Figure 1b, left, yellow filled circles), as well as the number of diffusion gradient directions (Supplementary Figure 1c, left, yellow filled circles). In other words, to evaluate connectomes for 1000 participants with LiFE, serial execution on a stand-alone desktop CPU would require multiple (>2) years of execution time.

To address the issue of slow execution time, we developed a GPU-accelerated version of LiFE's optimization algorithm: Subspace Barzilei-Borwein Non-Negative Least Squares (SBB-NNLS)[3] (Methods). Here, we specifically develop a CUDA implementation[4] for key computations involved in the SBB-NNLS algorithm. We addressed the algorithm's critical speed bottlenecks by splitting the computation for each voxel into distinct CUDA blocks and ensuring favorable memory access patterns into GPU kernels (Methods; Figure 1b). The GPU-implementation produces over a 100-fold improvement in connectome evaluation, in a manner that scaled robustly with connectome size, as described in the next section.

Over 100-fold speedup of connectome evaluation with GPUs

We tested the speedups obtained with the GPU-implementation of the LiFE algorithm (Methods) across 3 different datasets acquired with widely varying dMRI acquisition protocols and scanner configurations.

We tested for speedups, first, with a state-of-the-art diffusion MRI dataset (Dataset *H;* N_v=437,495, N_θ=270), from the HCP[2] database. We generated connectomes of 7 different sizes, ranging from 50,000 to 2 million fibers, with probabilistic tractography (Methods). The streamlines in these connectomes were then pruned with the CPU-implementation of LiFE (Supplementary Figure 1a, left, yellow filled circles, CPU-LiFE) as well with our GPU-

implementation of LiFE (Supplementary Figure. 1a, right, yellow open circles, GPU-LiFE), each for 500 iterations of the algorithm.

The GPU-implementation of LiFE produced significant speedups, as compared to the CPU-implementation. Runtimes were significantly lower for the GPU- compared to the CPU-implementation (ANOVA p=0.003; Supplementary Figure 1c, right, yellow open circles): speedups ranged from 62-fold (62x; 95% CI: [59.8, 63.4], across n=10 iterations) for a connectome with 50,000 fibers upto a maximum of 129-fold (129x; 95% CI: [128.8, 129]) for a connectome of 1.5 million fibers (Figure 1c, left, yellow filled circles).

Next, we evaluated these speedups by testing the GPU-implementation of LiFE on two other, independently acquired datasets. First, we optimized a dMRI dataset acquired in-house (Dataset *I;* N_v=116,468, N_θ=64, Methods). Again, we observe a maximum speedup of 124x (95% CI: [123.6, 124.4]) for a connectome of 1.5 million fibers (Figure 1c, left, black filled circles). Second, we optimized a dataset from Stanford University (Dataset *S;* N_v=247,969, N_θ=96, Methods), used as the benchmark in the original LiFE study[5] . Here, again, we observed a maximum speedup of over 155x (95% CI: [155.1, 155.2]) for a connectome of 1.5 million fibers (Figure 1c, left, blue filled circles).

Finally, to test how speedups scaled with the number of voxels (N_v) and directions (N_θ), we evaluated a connectome of 1 million fibers, while systematically varying N_v or N_θ, each, while keeping the other parameter at a constant value. For Dataset *H*, speedups increased gradually with the number of voxels (N_v), ranging from 98x for N_v=19,803 to >105x for N_v=437,495 (Figure 1c, middle, yellow filled circles; N_θ=270). With N_θ, speedups scaled up more dramatically, from 82x for N_θ=20 to >140x for N_θ=192 (Figure 1c, right, yellow filled circles; N_v=437,495); speedups decreased for very large N_θ (98x for N_θ=270; Discussion). Overall, speedups scaled fastest with number of diffusion directions, N_θ (speedup scaling

factor q_θ=22.8; Methods), followed by connectome size, N_f (q_f=18.2), and finally, spatial resolution N_v (q_v=4.6) (Supplementary Figure 1d). We repeated these same analyses with the two other datasets (*I* and *S*), and observed similar trends (Supplementary Figure 1b-d).

We also compared convergence times for the GPU-implementation of LiFE with two other state-of-the-art pruning algorithms, SIFT[6], and COMMIT2[7]. To ensure a fair comparison between GPU-accelerated LiFE and each of these methods, we pruned down a 1 million fiber connectome to comparable final streamline counts (to within 1%) for each speedup evaluation. Both GPU-accelerated LiFE and SIFT performed comparably (Supplementary Figure 1e, top panel), with a slight advantage for GPU-LiFE (Dataset *H*: speedup=1.3x; Dataset I: speedup=1.3x, Dataset *S*=1.8x). GPU-LiFE also performed comparably with COMMIT2. COMMIT2 outperformed GPU-LiFE for the smaller dataset (Dataset *I*: speedup=0.7x), whereas GPU-LiFE outperformed COMMIT2 for the two larger datasets (Dataset *S*: speedup=1.1x, Dataset *H*: speedup=2.2x) (Supplementary Figure 1e, bottom panel). Note that faster pruning times may be achieved for each method with more powerful hardware, for example, for SIFT and COMMIT2, by running on multi-core CPUs, and for GPU-LiFE, by running on multiple GPUs.

In summary, we obtained over 100x (up to 155x) speedups with our GPU implementation, as compared to the CPU implementation of the LiFE algorithm. Speedups scaled with increasing dataset size, in terms of all parameters tested: the number of voxels (N_v), the number of gradient directions (N_θ) and the initial connectome size (N_f). In other words, our GPU implementation accelerated LiFE's pruning algorithm sufficiently, to enable connectome evaluation at scale.

Regularized pruning yields more accurate connectomes

In their current form, streamline pruning algorithms do not explicitly encourage sparsity when estimating the fiber weight vector. As a consequence, weights may get split across identical

or closely similar fibers that contribute significantly to the underlying diffusion signal (Supplementary Figure 2a).

To illustrate this limitation, we generated a 1-million fiber connectome from a simulated dMRI signal, Dataset *M*[8], comprising almost entirely duplicated fibers (Methods). We then pruned this connectome with the state-of-the-art SIFT2[9] algorithm. Following pruning, the majority of duplicate fibers were retained, with nearly identical weights across each pair of duplicates (Supplementary Figure 2c, left). We quantified a "uniqueness index", based on the normalized difference of duplicate fiber weights; smaller values of this metric indicate a higher tendency to retain duplicate fibers ($\zeta_{\text{uniq}} = |w_1 - w_2|/|w_1 + w_2|$; Methods). Following SIFT2 pruning, ζ_{uniq} values were 0.0002 ± 0.0006 (median ± stderr), suggesting that duplicate fibers were largely retained even after pruning (Supplementary Figure 2b, top panel, x-axis).

We sought to address this limitation directly during LiFE's optimization by incorporating L1 regularization for the fiber weight vector ($\lambda \ \|\mathrm{w}\|_1$) to the objective function, producing sparser connectomes (Methods). We call this updated LiFE algorithm, implemented on GPUs with the regularized objective function, as "Regularized, Accelerated Linear Fascicle Evaluation" or ReAl-LiFE. We repeated the experiment by pruning duplicated fibers, both with LiFE's default algorithm as well as the ReAl-LiFE algorithm. ζ_{uniq} values for both LiFE and ReAl-LiFE (LiFE: 0.27 ± 0.0007, 95% CI: [0.267, 0.278], ReAl-LiFE: 0.45 ± 0.0007, 95% CI: [0.442, 0.466], across $\mathrm{N_f}$=500,000 fibers) were significantly higher than those for SIFT2 ($p<0.001$, Wilcoxon signed rank test). Specifically, ζ_{uniq} values for ReAl-LiFE (λ =0.05) were significantly higher than those for LiFE (Supplementary Figure 2b bottom, $p<0.001$, Wilcoxon signed rank test; effect size=0.207, Cohen's *d*) and remained fairly stable (or marginally increased) at higher λ values (Supplementary Figure 2d).

We repeated this experiment with an alternative approach for simulating duplicated fibers, by removing ~5% of the nodes from each end of every fiber and subsequently combining it with the original connectome (Methods). We then pruned this connectome with the modified ReAl-LiFE algorithm as well as SIFT2 and quantified the ζ_{uniq} values for each. With this approach, ζ_{uniq} for ReAl-LiFE was significantly higher compared to that for SIFT2, and comparable to that for LiFE (SIFT2: 0.0 ± 0.0, LiFE: 1 ± 0.0003, ReAl-LiFE (λ=0.05): 1 ± 0.0007; p<0.001, Wilcoxon signed rank test). In other words, among the three algorithms compared, ReAl-LiFE was the more successful at removing near-duplicate fibers in the data.

Next, we tested if ReAl-LiFE's regularized pruning algorithm would yield more accurate and reliable connectomes, as compared to LiFE, using real dMRI data.

First, we tested for the degree of "overfitting" with each algorithm (Supplementary Figure 3a; Methods). Briefly, we estimated cross-validation errors by estimating the connectome and pruning streamlines on the same dataset – a scenario that occurs commonly in situations when acquiring multiple independent dMRI datasets from the same participant is infeasible. A higher cross-validation error indicates a higher degree of "overfitting". We performed this cross-validation analysis with two datasets: Datasets *I* and S(*ET)*; Dataset S(*ET)* was an ensemble tractography dataset generated from the dMRI scan in Dataset *S* (Methods).

Pruning with ReAl-LiFE yielded lower cross-validation errors, on average, as compared to pruning with LiFE across a range of regularization parameter values (λ) (Supplementary Figure 3c, ReAl-LiFE: solid line and filled circles, vs LiFE: dashed horizontal line). The largest values of λ resulted in excessive pruning thereby yielding higher RMS errors for ReAl-LiFE (Supplementary Figure 3c). To enable a fair comparison, we computed the voxel-wise RMS cross-validation error of LiFE and ReAl-LiFE optimized connectomes at the value of λ that yielded matched fiber weights across the two evaluation methods, separately for

each dataset (Methods). The sum of fiber weights ($\|w\|_1$) estimated with ReAl-LiFE (Supplementary Figure 3b, filled circles) closely matched the sum of fiber weights as estimated with the unregularized LiFE algorithm (Supplementary Figure 3b, dashed horizontal line) near λ=0.01 (Supplementary Figure 3b, open square with dashed vertical line). At this value of λ, voxel-wise cross-validation error was systematically lower when fibers were pruned with ReAl-LiFE, as compared to LiFE, across all 3 datasets (Supplementary Figure 3d; $p<0.001$, Kolmogorov-Smirnov test comparing the LiFE and ReAl-LiFE RMSE error distributions; effect size: Dataset *S(ET)*, 0.065, Dataset I, 0.037).

Second, we tested for the consistency of evaluated connectomes with each algorithm (Supplementary Figure 4a; Methods). Briefly, we estimated cross-validation errors by estimating the connectome with one dataset and pruning its streamlines with a second dataset acquired from the same participant. A lower cross-validation error indicates a higher degree of consistency with connectome evaluation, across the two datasets, of the optimized connectomes. Again, voxel-wise cross-validation error was systematically lower when each connectome underwent streamline pruning with ReAl-LiFE, as compared to LiFE, across all 3 datasets (Supplementary Figure 4b; $p<0.001$, Kolmogorov-Smirnov test; effect size: Dataset *S(ET)*, 0.072, Dataset *I*, 0.038).

We also tested streamline pruning with alternative regularization approaches. Specifically, we tested whether L2 regularization would provide better cross-validation accuracies. First, we plotted cross-validated RMSE – obtained with the test for “overfitting” (Supplementary Figure 3c) – for the streamlines pruned each way (L1 or L2 regularization) as a function of the L1 norm of the weights, for different values of the regularization parameter (λ). Cross-validated RMSEs were typically lower for the L1-regularized connectome (Supplementary Figure 4c, filled circles) as compared to the L2-regularized connectome (Supplementary Figure 4c, open circles). Similarly, we plotted cross-validated RMSE for the L1 or L2 regularized connectomes as a function of the L2 norm of the weights for different λ values. In

this case, cross-validated RMSEs were uniformly lower for the L2-regularized connectome (Supplementary Figure 4d, open circles) as compared to the L1-regularized connectome (Supplementary Figure 4d, filled circles). In other words, the cross-validated RMSEs were lower for the respective norm (L1 or L2) that was penalized by the ReAl-LiFE objective.

Taken together, these results indicate that, as compared to the LiFE's default algorithm, the regularized objective function in ReAl-LiFE yielded connectomes that were both more accurate, in terms of their fit to the underlying diffusion signal, and more consistent, when evaluated across multiple datasets acquired from the same participant. Connectomes with desired sparsity characteristics (L1 or L2) could be generated with an appropriate choice of regularization term.

Behavioral predictions with structural features: Control analyses

To test the robustness of these behavioral score predictions based on ReAl-LiFE weights, we performed three control analyses. First, we considered the possibility that the superlative prediction accuracies could arise from mutual correlations among these behavioral scores. To address this, we replicated a previously published control analysis[10] by predicting a subset of 5 minimally correlated scores (Supplementary Data File 1, asterisk; Methods). Three out of these 5 scores were predicted significantly (Supplementary Figure 5b). Moreover, average prediction accuracy across these 5 scores (r_5=0.188±0.128; mean ± std), was comparable to the average prediction accuracy across all 60 scores (r_{60}=0.171±0.108).

Second, we controlled for confounds associated with motion parameters[11,12] by repeating the behavioral predictions after regressing out the effects of head motion from the behavioral scores (Methods). Following this control, behavioral score predictions continued to be strongly correlated with the original predictions (r=0.914, p<0.001, Supplementary Fig. 5c).

Third, we controlled for the possible biases in SVR-RFE algorithm with a random permutation test (Methods), generating a null distribution for each score prediction. With increasing number of RFE iterations for generating the null distribution (n = 1, 2, …,10), the number of significantly predicted scores increased from 9 to 14, monotonically (Supplementary Fig. 5d; p=0.05 threshold, Benjamini-Hochberg correction for multiple comparisons). These results indicate that more scores could be significantly predicted with a greater number of runs of the RFE algorithm, as estimates of the null distribution became progressively more robust.

Behavioral predictions with structural features: Univariate correlations

To analyze the anatomical features underlying these predictions, focusing on the "cognition" scores, we first identified the 5 cognition scores best predicted by ReAl-LiFE connection weights. These scores included: i) reading ability (ReadEng), ii) picture vocabulary (PicVocab), iii) delay discounting test for self-regulation (DDisc_AUC_40K), iv) spatial orientation (VSPLOT_TC), and v) sustained attention (SCPT_SEN). For each score, we identified the top 5 anatomical connections based on their beta weights in the prediction analysis. We then examined univariate correlations of each cognitive score with 3 different anatomical features associated with these connections: i) the number of fibers following ReAl-LiFE pruning, ii) fiber volume, and iii) fiber length (voxels intersected).

In general, stronger univariate correlations occurred between cognitive scores and the number of fibers following ReAl-LiFE pruning, than with average fiber volume or fiber length (median absolute r-value: number of fibers = 0.122, CI = [0.087, 0.161]; fiber volume = 0.041, CI = [0.022, 0.063]; fiber length = 0.054, CI = [0.022, 0.087]). Specifically, reading ability correlated significantly, and positively, with the number of fibers for connections between the left hemispheric supramarginal and inferior parietal gyrus (Supplementary Figure 5e, r=0.21, p=0.004). Moreover, reading ability also correlated significantly both with the number of fibers and fiber volume between the right hemispheric superior frontal gyrus

and frontal pole (number of fibers, r=0.19, p=0.006; fiber volume: r=0.15, p=0.039). These findings are consistent with the involvement of the left temporo-parietal cortex in language skills[13] and of the right prefrontal cortex in semantic and prosody processing[14]. Second, picture vocabulary scores correlated positively with the number of fibers connecting the left hemispheric supramarginal and superior parietal gyrus (Supplementary Figure 5e, r=0.25, p<0.001), as well as with fiber lengths of the connection between the right inferior frontal gyrus (pars orbitalis) and inferior parietal lobe, possibly reflecting the need for accurate phonological processing and accurate retrieval of pictorial representations from memory to successfully solve this task[15,16]. Third, delay discounting scores were positively correlated with the number of fibers for connections in the right inferior frontal cortex (r=0.27, p<0.001) as well as with the left medial orbital frontal cortex with the frontal pole (Supplementary Figure 5e, r=0.16, p=0.023), potentially reflecting the need for evaluation of delayed rewards[17,18]. Fourth, spatial orientation scores were significantly correlated with the number of fibers for multiple connections involving the superior parietal cortex (left superior parietal-supramarginal: r=0.16, p=0.027; left superior parietal-cuneus: r=0.18, p=0.011); these results are consistent with the well-documented role of the parietal cortex in computations involving spatial orientation[19,20]. No significant univariate correlations occurred between the top 5 anatomical features and scores on the spatial attention task. Full results, showing univariate correlations of each cognitive score with each of the three sets of features, are reported in Supplementary Data File 2.

Supplementary Information: Algorithms

GPU implementation of $\mathbf{y} = \mathbf{M} \cdot \mathbf{x}(\boldsymbol{\Phi}, \mathbf{D}, \mathbf{x})$

- Block size: 32
- Grid size: Number of voxels
- Each block handles one voxel. $\boldsymbol{\Phi}$ tensor is stored in COO format - entries $[\mathrm{a}, \mathrm{v}, \mathrm{f}, \mathrm{c}]$ where $\mathrm{a(n), v(n), f(n), c(n)}$ are the atom index, voxel index, fiber index and the corresponding value at those indices respectively. In the following, $[\mathrm{a_i f_i, c_i}]$ denote the corresponding entries for voxel i.
- N_ω, the number of diffusion directions handled by each thread is pre-calculated. It is equal to the (number of diffusion directions)$/32$.
- $\mathrm{N_v(i)}$ refers to the number of $\boldsymbol{\Phi}$ entries for voxel i.

1. Set $\mathrm{index} = 1$.
2. In block i, read up to $\mathrm{N_b} <= 32$ entries of voxel i from the $\boldsymbol{\Phi}$ tensor. In thread j, read $\mathrm{a_i(j), x(f_i(j)), c_i(j)}$ into thread local memory.
3. Broadcast $\mathrm{a_i(j), x(f_i(j)), c_i(j)}$ from thread $\mathrm{j} = \mathrm{index \bmod 32}$ to all threads into local variables $\mathrm{a, x, c}$.
4. In thread j, initialize local memory $\mathrm{y_l(q)} = 0$ where $\mathrm{q} = 1$ to N_ω.
5. If $\mathrm{x} = 0$, go to Step (6). Else, in thread j

for $\mathrm{q} = 1$ to N_ω **do**
    $\mathrm{y_l(q) = y_l(q) + D(a, j + q * 32) * x * c}$
end for

6. $\mathrm{index} = \mathrm{index} + 1$, if $(\mathrm{index \bmod 32}) < \mathrm{N_b}$, go to Step (3). Else continue.
7. $\mathrm{index} = \mathrm{index} + 32$; if $\mathrm{index} < \mathrm{N_v(i)}$, go to Step (2). Else continue.
8. Write the results back to global memory. In block i, thread j

for $\mathrm{q} = 1$ to N_ω **do**
    $\mathrm{y(i, j + q * 32) = y_l(q)}$
end for

Supplementary Algorithm 1. **GPU implementation of matrix multiplications of the form y = M x.** Pseudocode describing the GPU implementation of multiplications of the form y = M x.

GPU implementation of $\mathbf{x} = \mathbf{M}^\mathbf{T} \cdot \mathbf{y}(\boldsymbol{\Phi}, \mathbf{D}, \mathbf{y})$

- Block size: 32
- Grid size: Number of voxels (other notations as in algorithm S1).

1. In block i, read voxel data from global memory into local memory y_l. In thread j
for $\mathrm{q} = 1$ to N_ω **do**
    $\mathrm{y_l(q) = y(i, j + q * 32)}$
end for
2. Set $\mathrm{index} = 1$.
3. In block i, read up to $\mathrm{N_b} <= 32$ entries of voxel i from the $\boldsymbol{\Phi}$ tensor. In thread j, read $\mathrm{a_i(j), f_i(j), c_i(j)}$ into thread local memory.
4. Broadcast $\mathrm{a_i(j), c_i(j)}$ from thread $\mathrm{j = index \bmod 32}$ to all threads into local variables $\mathrm{a, c}$.
5. In thread j, initialize local memory $\mathrm{res} = 0$.
6. In thread j
for $\mathrm{q} = 1$ to N_ω **do**
    $\mathrm{res = res + D(a, j + q * 32) * y_l(q) * c}$
end for
7. Compute the sum of res local variables using warp shuffle down reduction, the final sum being stored in res of thread 0.
8. In thread 0, store the computed sum in shared variable, $\mathrm{s_x(index \bmod 32) = res}$.
9. $\mathrm{index = index + 1}$, if $\mathrm{(index \bmod 32) < N_b}$, go to Step (4). Else continue.
10. Write the results back to global memory. In thread $\mathrm{j < N_b}$,
$\mathrm{atomic\_add(x(f_i(j)), s_x(j))}$
where $\mathrm{atomic\_add(a, b)}$ adds b atomically to a.
11. $\mathrm{index = index + 32}$, if $\mathrm{index < N_v(i)}$, go to Step (3). Else return.

Supplementary Algorithm 2. **GPU implementation of matrix multiplications of the form $x = M^T y$.** Pseudocode describing the GPU implementation of multiplications of the form $x = M^T y$.

Supplementary Information: Tables

**Model**	**Parameter name**	**Parameter value**
**Inter-axonal (Tensor model)**	T2 relaxation	110 ms
	Axial diffusivity (d1)	0.0012 cm^2s^{-1}
	Radial diffusivity (d2)	0.003 cm^2s^{-1}
**Intra-axonal (stick model)**	T2 relaxation	110 ms
	Diffusivity	0.0012 cm^2s^{-1}
**Grey matter (GM; ball model)**	T2 relaxation	80 ms
	Diffusivity	0.001 cm^2s^{-1}
**Cerebrospinal fluid (CSF; ball model)**	T2 relaxation	2500 ms
	Diffusivity	0.002 cm^2s^{-1}
**Artifacts**	Noise	Gaussian; variance 251
	Eddy currents	None
	Motion (random)	Rotation: [x,y,z] = [0,0,4]
		Translation: [x,y,z] = [2,0,0]
	Gibbs ringing	None
	Aliasing	None
	Distortion	Yes
	Spikes	None

Supplementary Table 1. Model parameters used for generating simulated Dataset *M*.

Model parameter values used to simulate artificial diffusion MRI data using Fiberfox[8].

100206	105923	115724	146937	153227	160729	186141	449753	523032	571548
100307	106016	116524	147636	153631	160830	192035	453441	524135	573249
100408	106824	117122	147737	153732	160931	193441	454140	529953	573451
100610	107321	118225	148032	153833	161327	194746	461743	530635	578158
101006	107725	118528	148133	154229	161630	199251	462139	531536	580751
101309	108020	122822	148335	154431	161731	200614	465852	540436	581349
101410	108121	123824	148840	154835	162026	205119	467351	541640	581450
101915	108222	125525	149236	155231	162329	209935	479762	545345	583858
102008	108828	130013	149842	155635	162733	217429	480141	548250	635245
102109	110411	132017	150019	156031	163432	228434	486759	550439	673455
102513	111312	134425	150524	156233	163836	249947	495255	552241	713239
102614	111716	135124	150928	156334	164636	325129	497865	555954	715041
102816	112314	135528	151223	157336	166640	341834	506234	557857	735148
103111	112920	136227	151425	157437	168240	394956	512835	558657	792766
103414	113619	138534	151627	157942	168341	414229	516742	559457	792867
103515	113922	140925	151829	158035	171330	421226	517239	561444	818859
103818	114318	145834	152225	159239	173132	424939	518746	561949	856463
104012	114419	146331	152427	159340	173233	433839	519647	567052	885975
105014	115219	146634	153025	159441	179952	445543	520228	570243	889579
105115	115320	146735	153126	159744	185139	448347	522434	571144	919966

Supplementary Table 2. List of participant IDs from the HCP database.

Table showing the identifiers for the 200 participants from the HCP database whose behavioral scores and diffusion MRI data was used for prediction analyses. Subject indices marked in blue correspond to those employed in the test-retest reliability analysis (Figs. 4a-d, main text).

	Most consistent	Least consistent
1	(Left) Caudal middle frontal – Pars opercularis	(Right) Middle temporal – Pericalcarine
2	(Right) Caudal anterior cingulate – middle orbitofrontal	(Left) Middle temporal – Superior temporal
3	(Right) Entorhinal – Precuneus	(Left) Posterior cingulate – Superior parietal
4	(Right) Superior frontal – Supramarginal	(Left) Middle orbitofrontal – Insula
5	(Left) Inferior parietal – Isthmus of the cingulate cortex	(Right) Lingual – Middle temporal
6	(Left) Lateral occipital – Rostral middle frontal	(Left) bank of the superior temporal sulcus – Inferior parietal
7	(Left) Inferior parietal – Pars opercularis	(Right) pericalcarine – Superior parietal
8	(Left) Inferior parietal – Inferior temporal	(Right) lateral occipital – Middle temporal
9	(Left) Precuneus – Superior frontal	(Left) Fusiform – isthmus of the cingulate cortex
10	(Left) Caudal middle frontal - Supramarginal	(Left) isthmus of the cingulate cortex – posterior cingulate

Supplementary Table 3. List of most consistent and least consistent connections.

List of top 10 connections that exhibited the highest (first column, decreasing order) and lowest (second column, increasing order) reliability indices (ϕ, see text for details).

Supplementary Information: Figures

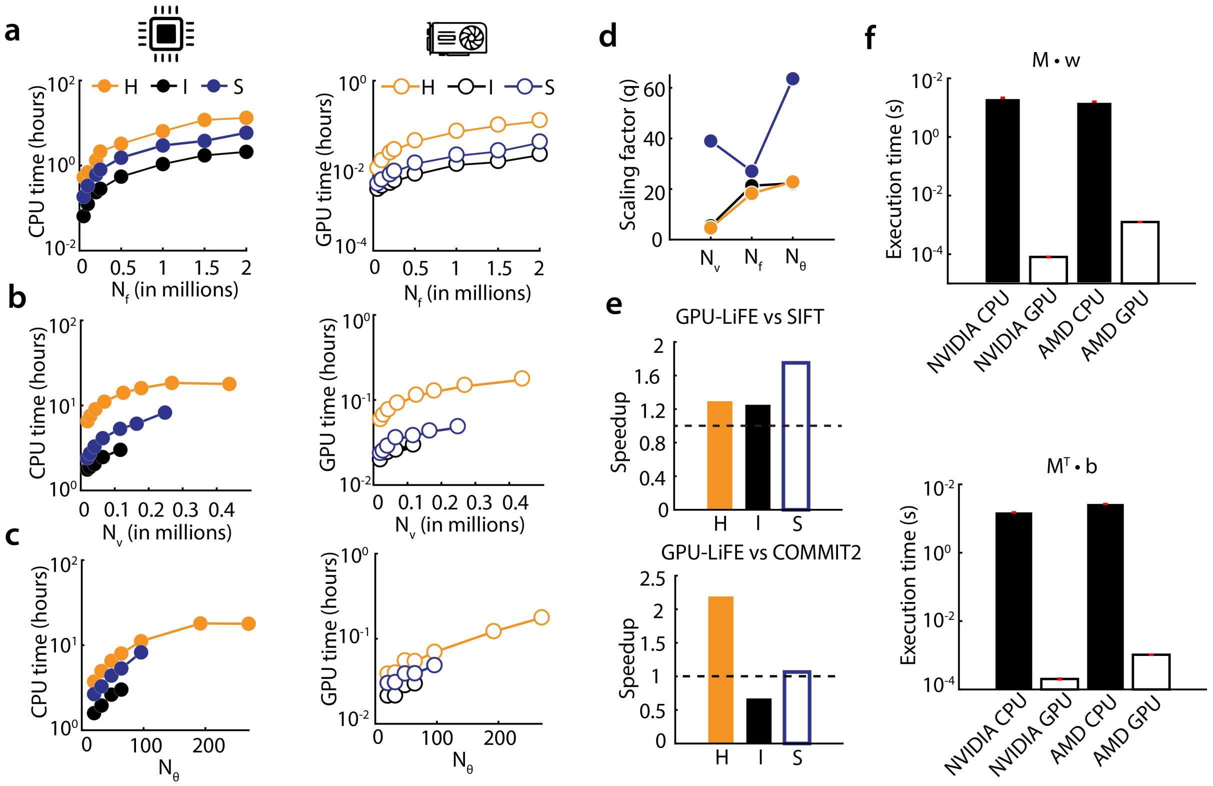

Supplementary Figure 1. Execution times for the CPU- and GPU- implementations of LiFE and speedups on the AMD GPU. a. (Left) Execution time (in hours) for 500 iterations of the CPU implementation of LiFE (CPU time, y-axis) as a function of the number of fibers N_f in each connectome (x-axis) for Datasets *H* (yellow), *I* (black) and *S* (blue), respectively. Filled circles: Connectome sizes tested. (Right) Execution time (in hours) for 500 iterations of the GPU implementation of LiFE (GPU time, y-axis) as a function of the number of fibers N_f (x-axis), for the same 3 datasets. Other conventions are as in the left panel. **b.** Same as in panel *a* but showing the execution times for 500 iterations of the CPU-implementation of LiFE (left) and for the GPU implementation of LiFE (right) as a function of the number of voxels N_v in each diffusion MRI volume (x-axis). All other conventions are as in panel *a*. **c.** Same as in panel *a* but showing the execution times for 500 iterations of the CPU-implementation of LiFE (left) and the GPU implementation of LiFE (right) as a function of the number of gradient directions N_θ for each diffusion MRI scan. All other conventions are as in

panel *a*. **d.** Speedup scaling factor (q) computed for each of the three parameters – N_v, N_f and N_θ, for Datasets H (yellow), I (black) and S (blue), respectively. **e.** Speedup of GPU implementation of LiFE over the SIFT method[6] (top) and over the COMMIT2 method[7] (bottom) for Datasets *H* (yellow), *I* (black) and *S* (blue). **f.** Average execution times across 10 runs for Algorithms 1 (**M.w**, left panel) and 2 ($\mathbf{M^T.b}$, right panel) on NVIDIA and AMD GPUs (open bars) and desktop CPUs (closed bars). NVIDIA GPU and AMD GPU refer to run times on the NVIDIA GeForce GTX 1080 Ti GPU and the AMD Radeon RX 580 GPU, respectively, whereas NVIDIA CPU and AMD CPU refer to run times on the CPUs in the desktop workstations on which these respective GPUs were installed (see Hardware Specifications in Methods). Error bars indicate the standard error of the mean.

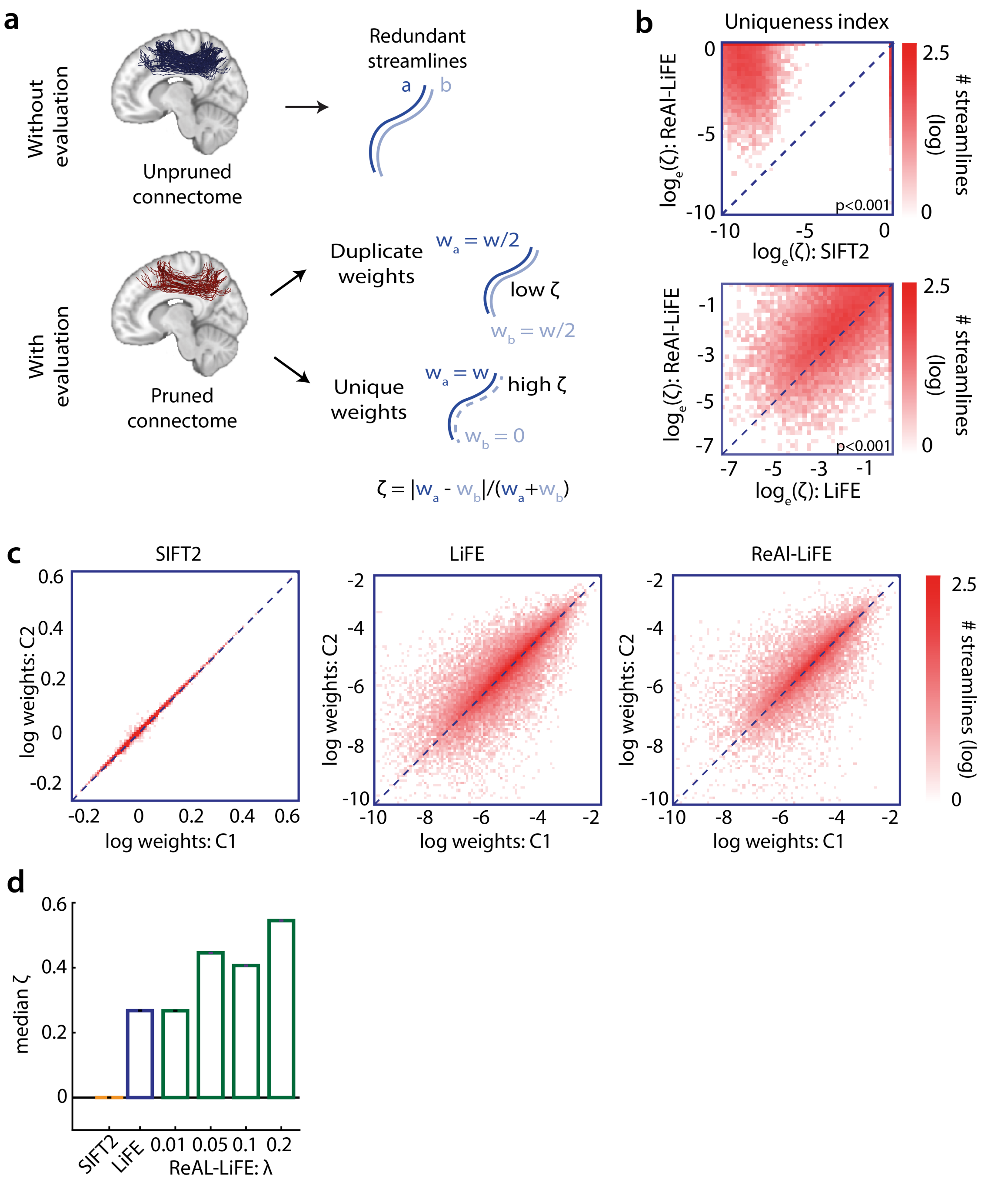

Supplementary Figure 2. Testing for weight duplication. a. (Top) Schematic showing an unpruned fascicle (left) containing two near-identical copies, *a* and *b*, of a representative fiber (right). (Bottom) Connectome evaluation can yield one of two extreme outcomes: each copy of the fiber is assigned an equal weight (duplicate weights, top), or the weight is assigned to only one of the two copies (unique weights, bottom); the latter is the desirable outcome. ζ: uniqueness index, computed as the normalized difference between the fiber

weights across copies; a higher value of ζ indicates better removal of duplicate fibers. **b.** (Top) Two-dimensional distribution of uniqueness index values after pruning a highly redundant 1-million fiber connectome with the proposed algorithm, ReAl-LiFE (Regularized, Accelerated, Linear Fascicle Evaluation; y-axis, $\lambda = 0.05$) versus pruning with an established algorithm, SIFT2 (x-axis). Deeper shades: higher proportions; blue dashed diagonal line: line of equality. (Bottom) Same as in the top panel but showing the distribution of uniqueness index values after pruning with ReAl-LiFE (y-axis, $\lambda = 0.05$) versus the original (unregularized) LiFE (x-axis) algorithm. Other conventions are the same as in the top panel. Wilcoxon signed-rank test, ***$p<0.001$ (top and bottom). **c.** Distribution of fiber weights across two near-identical halves C1 (0.5 million fibers, x-axis) and C2 (0.5 million fibers, y-axis) (Methods) of a 1 million fiber connectome generated from Dataset *M*, after pruning with SIFT2 (left), LiFE (middle) and ReAl-LiFE (right). Deeper shades indicate a greater proportion of fibers. Diagonal line: line of equality. **d.** Median uniqueness index of fiber weights (ζ) following pruning with SIFT (yellow bar), LiFE (blue bar) and ReAl-LiFE (green bars), for varying regularization penalty values λ. Uniqueness index values for SIFT are close to zero. Error bars indicate the standard error of the median.

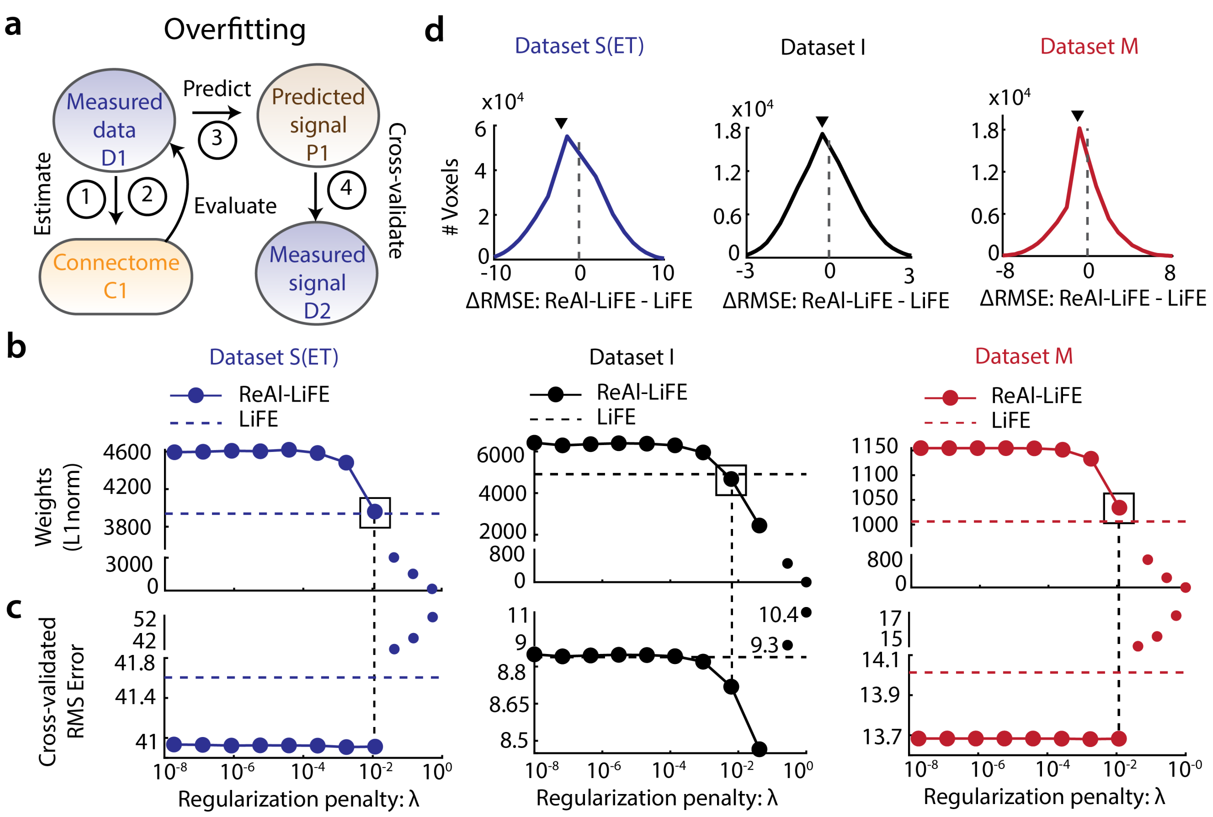

Supplementary Figure 3. Regularized pruning with ReAl-LiFE limits overfitting. a. Schematic of the procedure for evaluating model overfitting. Briefly, overfitting was measured by evaluating the model on the same diffusion MRI dataset used to generate the connectome (D1) and comparing the predicted signal (P1) against an independently acquired diffusion MRI dataset (D2) from the same participant (see text for details). **b.** (Left) L1 norm of fiber weights for a 1.6-million fiber, whole brain connectome generated with Dataset *S (ET*/ensemble tractography) pruned with ReAl-LiFE (blue filled circles and solid line) for various values of the regularization parameter λ. Blue dashed horizontal line: L1 norm of fiber weights for a 0.8 million fiber connectome pruned with LiFE. Open black square and dashed vertical line: λ corresponding to the matched L1 norm of fiber weights across the two algorithms. (Middle) Same as in the left panel but for Dataset *I*. (Right) Same as in the left panel but for Dataset *M*. Other conventions in middle and right panels are the same as in the left panel. For all panels, the y-axis is split into two halves to better visualize the variation in fiber weight L1 norms with λ. **c.** Same as in panel *b* but showing the cross-validated RMSE (root mean squared error) as a function of the regularization parameter λ for

Datasets *S(ET)* (left), *I* (middle) and *M* (right). Other conventions are the same as in panel *b*. For all panels, the y-axis is split into two halves to better visualize the variation in cross-validated RMSE with λ. Note that cross-validated RMSE for ReAl-LiFE is typically lower than that for LiFE for λ values that yield comparably sparse connectomes across both approaches (black squares in panel *b*). **d.** (Left) Overfitting-based distribution, across voxels, of differences in cross-validated RMSE between ReAl-LiFE and LiFE ($\Delta RMSE = RMSE_{ReAl\text{-}LiFE} - RMSE_{LiFE}$, x-axis) for Dataset *S(ET)* (same dataset as in panel *b*; λ=0.01 corresponding to black square in panel *b*, left). Black inverted triangle: mean; dashed grey vertical line: ΔRMSE=0. (Middle) Same as in the left panel but showing ΔRMSE for Dataset *I* (λ=0.006 corresponding to black square in panel *b*, middle). (Right) Same as in the left panel but showing ΔRMSE for Dataset *M* (λ=0.01 corresponding to black square in panel *b*, right). Other conventions in middle and right panels are the same as in the left panel. Error distributions were compared using the Kolmogorov-Smirnov test; ***$p<0.001$ for all three datasets.

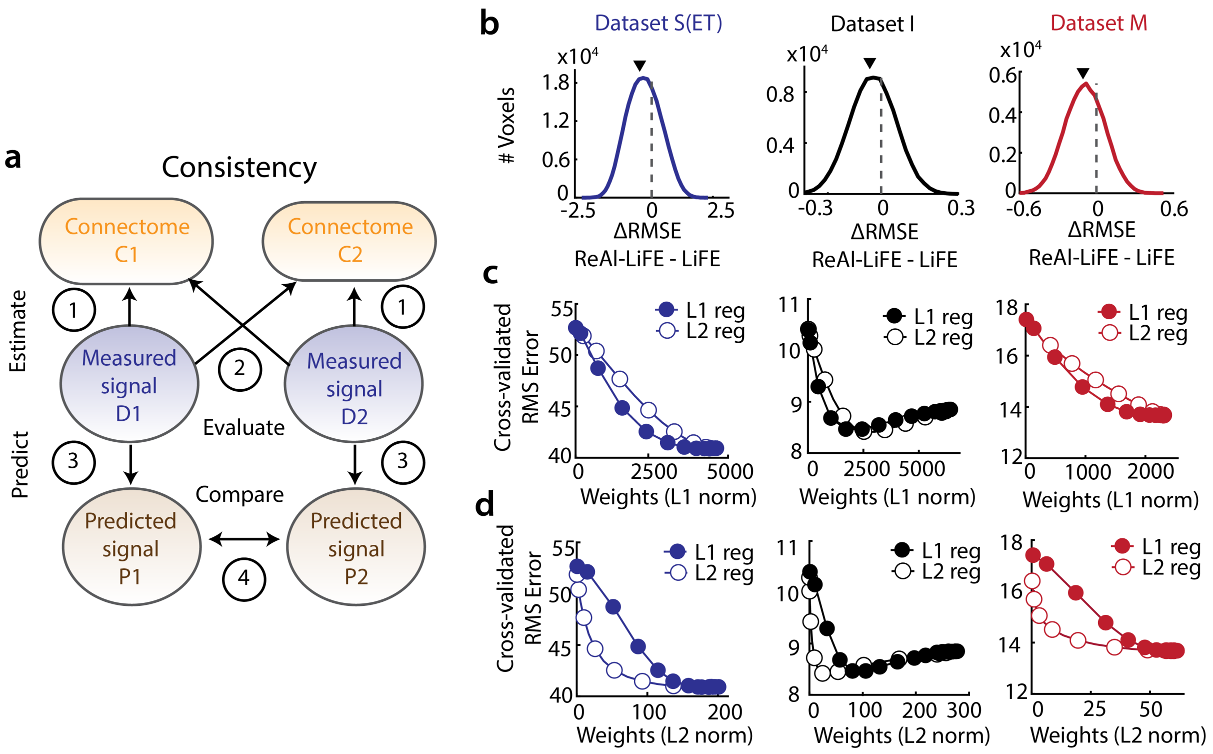

Supplementary Figure 4. Regularized pruning with ReAl-LiFE improves consistency. **a.** Schematic of the procedure for evaluating model consistency. Briefly, consistency was measured by evaluating the model on two independently acquired diffusion MRI datasets from the same participant (D1 and D2), and comparing the predicted signals (P1 and P2) across the two datasets (see text for details). **b.** (Left) Consistency-based distribution, across voxels, of differences in cross-validated RMSE between ReAl-LiFE and LiFE ($\Delta RMSE = RMSE_{ReAl\text{-}LiFE} - RMSE_{LiFE}$, x-axis), for Dataset *S(ET)*. Other conventions are the same as in panel Supplementary Figure 3d, left. (Middle) Same as in the left panel, but showing ΔRMSE for Dataset *I.* (Right). Same as in the left panel, but showing ΔRMSE for Dataset *M.* Other conventions in middle and right panels are the same as panels Supplementary Figure 3d, middle and right, respectively. Error distributions were compared using the Kolmogorov-Smirnov test; ***p<0.001 for all three datasets. **c.** (Left) Cross-validated RMSE (sum across voxels, y-axis) plotted against the L1 norm of fiber weights (x-axis) for a connectome generated with Dataset *S(ET)* (same as in Supplementary Figure 3b, left) and pruned with ReAl-LiFE (λ=0.01) using either L1 regularization (filled circles) or L2

regularization (open circles). Circles: Cross-validated RMSE for each λ value tested ([1e-8, 1]). For the L2 regularization results, only every alternate point is shown, for clarity. (Middle) Same as in the left panel but for Dataset *I*. (Right) Same as in the left panel but for Dataset *M*. Other conventions in middle and right panels are the same as in the left panel. **d.** (Left) Same as in panel *c*, left, but showing cross-validated RMSE (sum across voxels, y-axis) plotted against the L2 norm of fiber weights (x-axis) for Dataset *S(ET).* (Middle) Same as in the left panel, but for Dataset *I.* (Right) Same as in the left panel, but for Dataset *M.* Other conventions in middle and right panels are the same as in panel *c*, middle and right panels, respectively.

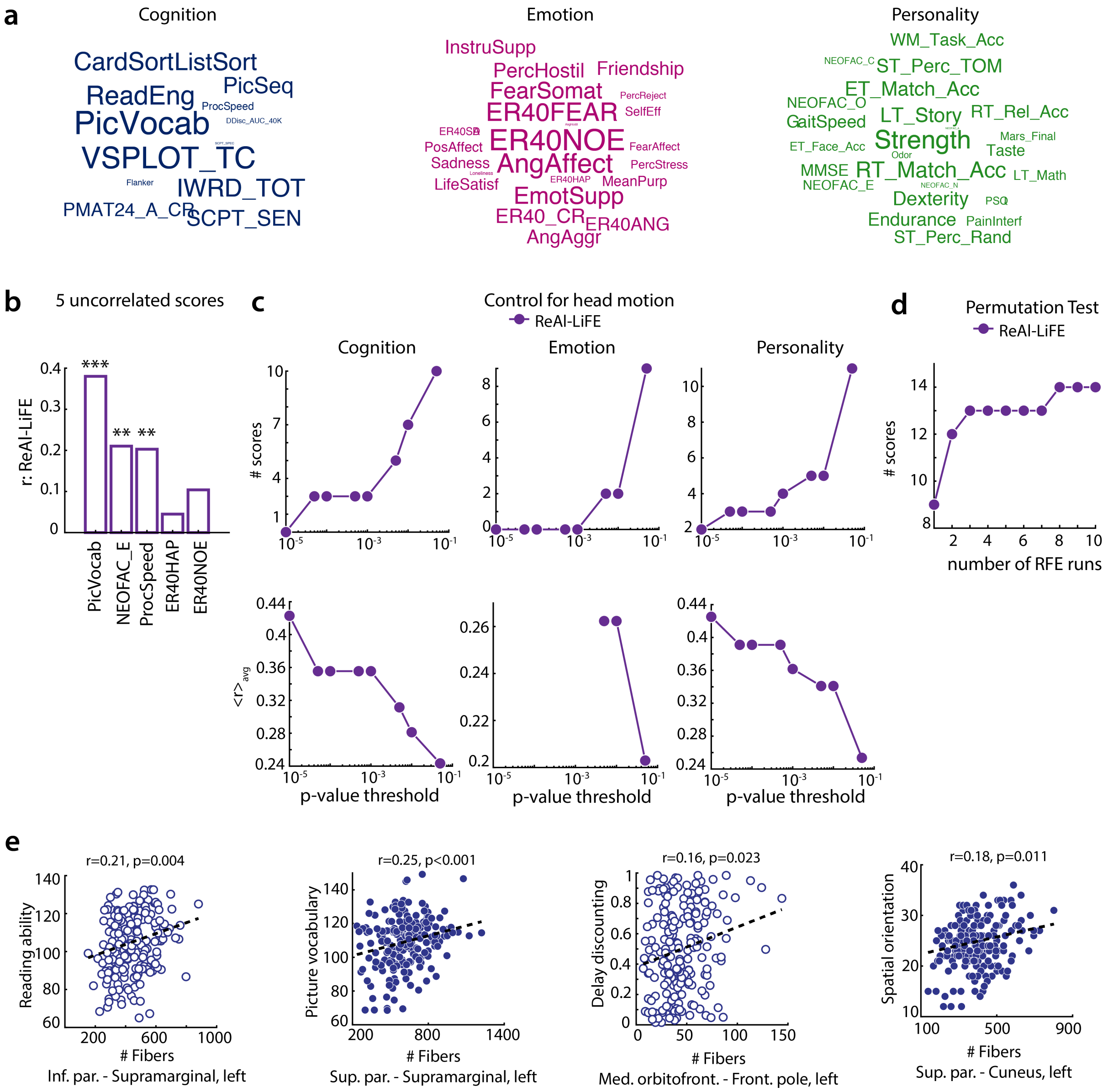

Supplementary Figure 5. **Analyzing structure-behavior relationships – Control analyses and univariate correlations. a.** Word clouds showing the different behavioral scores from the cognition (left), emotion (middle) and personality (right) category, sized based on their prediction accuracy values using the number of fibers. Larger words indicate better predicted scores. **b.** Correlation between the observed and predicted behavioral scores for 5 minimally correlated scores (Methods; predictions based on ReAl-LiFE connection weights; *** p<0.001, ** 0.001, *<p<0.01). **c.** Same as in Fig. 5c-e (main text) but following control for head motion (Methods). Other conventions as in Fig. 5c-e (main text). Prediction accuracies (r-values) were similar to those in the original analysis (compare with

Figs. 5c-e). **d.** Number of significantly predicted behavioral scores (p=0.05 threshold, Benjamini-Hochberg correction) following a random permutation test (y-axis) as a function of the number of RFE runs for generating the null distribution of prediction accuracies (x-axis; Methods; predictions based on ReAL-LiFE connection weights). Error bars: Standard error of the mean across all combinations of the respective RFE runs. Most error bars are smaller than the sizes of the respective symbols. **e.** Univariate correlations (based on robust correlation) between the number of fibers in ReAl-LiFE pruned tracts (x-axis) and best predicted cognitive scores (from left to right): reading ability (ReadEng; r=0.21, **p=0.004), picture vocabulary (PicVocab; r=0.25, ***p<0.001), delay discounting (DDisc_AUC_40K; r=0.16, **p=0.023), and spatial orientation (VSPLOT_TC; r=0.18, p=0.011). Each point corresponds to an individual participant (n=200). Dashed black line: Line of best fit.

Supplementary References

1. Caiafa, C. F. & Pestilli, F. Multidimensional encoding of brain connectomes. *Sci. Rep.* **7**, (2017).
2. Van Essen, D. C. *et al.* The Human Connectome Project: A data acquisition perspective. *NeuroImage* **62**, 2222–2231 (2012).
3. Kim, D., Sra, S. & Dhillon, I. S. A non-monotonic method for large-scale non-negative least squares. *Optim. Methods Softw.* **28**, 1012–1039 (2013).
4. NVIDIA. *Cuda C Programming Guide*. *Programming Guides* (2015).
5. Pestilli, F., Yeatman, J. D., Rokem, A., Kay, K. N. & Wandell, B. A. Evaluation and statistical inference for human connectomes. *Nat. Methods* **11**, 1058–1063 (2014).
6. Smith, R. E., Tournier, J. D., Calamante, F. & Connelly, A. SIFT: Spherical-deconvolution informed filtering of tractograms. *Neuroimage* **67**, 298–312 (2013).
7. Schiavi, S. *et al.* A new method for accurate in vivo mapping of human brain connections using microstructural and anatomical information. *Sci. Adv.* **6**, (2020).
8. Maier-Hein, K. H. *et al.* The challenge of mapping the human connectome based on diffusion tractography. *Nat. Commun.* **8**, (2017).
9. Smith, R. E., Tournier, J. D., Calamante, F. & Connelly, A. SIFT2: Enabling dense quantitative assessment of brain white matter connectivity using streamlines tractography. *Neuroimage* **119**, 338–351 (2015).
10. Kong, R. *et al.* Spatial topography of individual-specific cortical networks predicts human cognition, personality, and emotion. *Cereb. Cortex* **29**, 2533–2551 (2019).
11. Havsteen, I. *et al.* Are movement artifacts in magnetic resonance imaging a real problem?- A narrative review. *Frontiers in Neurology* **8**, (2017).
12. Baum, G. L. *et al.* The impact of in-scanner head motion on structural connectivity derived from diffusion MRI. *Neuroimage* **173**, 275–286 (2018).
13. Hertrich, I., Dietrich, S. & Ackermann, H. The Margins of the Language Network in the Brain. *Frontiers in Communication* **5**, 93 (2020).
14. Hertrich, I., Dietrich, S., Blum, C. & Ackermann, H. The role of the dorsolateral prefrontal cortex for speech and language processing. *Frontiers in Human Neuroscience* **15**, 217 (2021).
15. Barbey, A. K., Koenigs, M. & Grafman, J. Dorsolateral prefrontal contributions to human

working memory. *Cortex* **49**, 1195–1205 (2013).

16. Deschamps, I., Baum, S. R. & Gracco, V. L. On the role of the supramarginal gyrus in phonological processing and verbal working memory: Evidence from rTMS studies. *Neuropsychologia* **53**, 39–46 (2014).

17. Rogers, R. D. *et al.* Distinct portions of anterior cingulate cortex and medial prefrontal cortex are activated by reward processing in separable phases of decision-making cognition. *Biol. Psychiatry* **55**, 594–602 (2004).

18. Rolls, E. T. The Orbitofrontal Cortex and Reward. *Cereb. Cortex* **10**, 284–294 (2000).

19. Bisley, J. W. & Goldberg, M. E. Attention, intention, and priority in the parietal lobe. *Annu. Rev. Neurosci.* **33**, 1–21 (2010).

20. Freedman, D. J. & Ibos, G. An Integrative Framework for Sensory, Motor, and Cognitive Functions of the Posterior Parietal Cortex. *Neuron* **97**, 1219–1234 (2018).
