# Supplementary material for: GPU-accelerated connectome discovery at scale
Source: Nat Comput Sci. 2022 May 30;2(5):298–306. doi: 10.1038/s43588-022-00250-z (PMC10766542; doi:10.1038/s43588-022-00250-z)
Supplement: Supplementary file 2 — Reporting Summary [file 43588_2022_250_MOESM2_ESM.pdf]

Corresponding author(s): Devarajan Sridharan

Last updated by author(s): Apr 15, 2022

Reporting Summary

Nature Research wishes to improve the reproducibility of the work that we publish. This form provides structure for consistency and transparency in reporting. For further information on Nature Research policies, see our Editorial Policies and the Editorial Policy Checklist.

Statistics

For all statistical analyses, confirm that the following items are present in the figure legend, table legend, main text, or Methods section.

n/a	Confirmed	
☐	☒	The exact sample size (*n*) for each experimental group/condition, given as a discrete number and unit of measurement
☐	☒	A statement on whether measurements were taken from distinct samples or whether the same sample was measured repeatedly
☐	☒	The statistical test(s) used AND whether they are one- or two-sided<br>*Only common tests should be described solely by name; describe more complex techniques in the Methods section.*
☐	☒	A description of all covariates tested
☐	☒	A description of any assumptions or corrections, such as tests of normality and adjustment for multiple comparisons
☐	☒	A full description of the statistical parameters including central tendency (e.g. means) or other basic estimates (e.g. regression coefficient) AND variation (e.g. standard deviation) or associated estimates of uncertainty (e.g. confidence intervals)
☐	☒	For null hypothesis testing, the test statistic (e.g. *F*, *t*, *r*) with confidence intervals, effect sizes, degrees of freedom and *P* value noted<br>*Give P values as exact values whenever suitable.*
☒	☐	For Bayesian analysis, information on the choice of priors and Markov chain Monte Carlo settings
☒	☐	For hierarchical and complex designs, identification of the appropriate level for tests and full reporting of outcomes
☐	☒	Estimates of effect sizes (e.g. Cohen's *d*, Pearson's *r*), indicating how they were calculated

Our web collection on statistics for biologists contains articles on many of the points above.

Software and code

Policy information about availability of computer code

Data collection	Dataset I was acquired at the HealthCare Global Hospital, Bangalore.
Data analysis	Dataset I was preprocessed using Vistasoft 2017 (https://github.com/vistalab/vistasoft).<br>Dataset S was available in preprocessed format. Preprocessing was performed using Vistasoft 2017<br>Dataset M was preprocessed using Mrtrix3 and FSL.<br>Dataset H and all data used for behavioral predictions, we used minimally preprocessed data as part of the HCP S1200 release.<br>For all datasets, tractography was performed using MRtrix3 and cortical parcellations were obtained using FreeSurfer. All analyses were carried out using custom code written in MATLAB2017b. Code for the ReAl-LiFE algorithm is available at 10.24433/CO.5578739.v1.

For manuscripts utilizing custom algorithms or software that are central to the research but not yet described in published literature, software must be made available to editors and reviewers. We strongly encourage code deposition in a community repository (e.g. GitHub). See the Nature Research guidelines for submitting code & software for further information.

Data

Policy information about availability of data

All manuscripts must include a data availability statement. This statement should provide the following information, where applicable:

- Accession codes, unique identifiers, or web links for publicly available datasets
- A list of figures that have associated raw data
- A description of any restrictions on data availability

Dataset I was acquired at the HealthCare Global Hospital, Bangalore.
Dataset S was acquired at Stanford's Center for Cognitive and Neurobiological Imaging, and is publicly available at doi:10.5967/K8X63JTX.

Dataset M is a synthetic dataset generated as part of the ISMRM Tractography Challenge 2015, and is available at http://tractometer.org/ismrm_2015_challenge/data.
Dataset H and all data used for behavioral predictions are part of theWU-Minn HCP Consortium S1200 Release, and are publicly accessible at https://www.humanconnectome.org.

Data availability
The datasets generated during and/or analyzed during the current study are available in the repositories linked below:
Dataset I: 10.6084/m9.figshare.13491024
Dataset S/S(ET): doi:10.5967/K8X63JTX.
Dataset H: https://www.humanconnectome.org/study/hcp-young-adult
Dataset M: http://www.tractometer.org/ismrm_2015_challenge/
Source Data for Figure 1 and Figure 2 are available with this manuscript.

Field-specific reporting

Please select the one below that is the best fit for your research. If you are not sure, read the appropriate sections before making your selection.

☒ Life sciences ☐ Behavioural & social sciences ☐ Ecological, evolutionary & environmental sciences

For a reference copy of the document with all sections, see nature.com/documents/nr-reporting-summary-flat.pdf

Life sciences study design

All studies must disclose on these points even when the disclosure is negative.

Sample size	Power analysis for sample size estimation was not performed. Datasets I, H, S and M are drawn from diverse databases and correspond each to scans from individual subjects; these were meant to illustrate ReAl-LiFE speedups and features. For behavioral predictions, data for 200 participants were drawn from the HCP database in chronological order, after matching for age and gender. Sample sizes (>100) are typical for studies in this domain and similar sample sizes have been routinely employed in previous studies for establishing brain-behavior relationships. A systematic analysis of the effect of sample sizes on brain-wide association studies is available at: https://doi.org/10.1038/s41586-022-04492-9
Data exclusions	Because tractography is a time consuming step, dMRI scans for 200 subjects in the HCP database were analyzed for behavioral predictions. These were drawn in chronological order from among the 1200 subjects' data available in the database, matching for age and gender , in a manner agnostic to prediction results.
Replication	We confirmed that all key results pertaining to GPU acceleration and regularization were replicated across three independently acquired datasets (Datasets I, S and H).
Randomization	No experimental groupings were used in this study.
Blinding	Blinding is not relevant because no grouping was performed.

Reporting for specific materials, systems and methods

We require information from authors about some types of materials, experimental systems and methods used in many studies. Here, indicate whether each material, system or method listed is relevant to your study. If you are not sure if a list item applies to your research, read the appropriate section before selecting a response.

Materials & experimental systems

n/a	Involved in the study	
☒	☐	Antibodies
☒	☐	Eukaryotic cell lines
☒	☐	Palaeontology and archaeology
☒	☐	Animals and other organisms
☐	☒	Human research participants
☒	☐	Clinical data
☒	☐	Dual use research of concern

Methods

n/a	Involved in the study	
☒	☐	ChIP-seq
☒	☐	Flow cytometry
☐	☒	MRI-based neuroimaging

Human research participants

Policy information about studies involving human research participants

Population characteristics	Dataset I: Female, 26-29 yrs.

Population characteristics	Dataset S: Male, 37-39 yrs.<br>Dataset H: Male, 26-30 yrs.<br>Dataset M: synthetic data generated from one HCP dataset, male, 26-30 yrs.<br>Data for behavioral predictions: 100 females, 22-37 yrs.
Recruitment	Dataset I: Acquired as part of a previous study (Sreenivasan and Sridharan, PNAS, 2019).<br>Dataset S: Information can be found at doi:10.5967/K8X63JTX.<br>Dataset M: Information can be found at http://tractometer.org/ismrm_2015_challenge/data.<br>Dataset H and all data used for behavioral predictions are part of WU-Minn HCP Consortium S1200 Release. Information for these datasets can be found at https://www.humanconnectome.org/study/hcp-young-adult/project-protocol/recruitment.
Ethics oversight	Institute Human Ethics Committee, Indian Institute of Science, Bangalore.

Note that full information on the approval of the study protocol must also be provided in the manuscript.

Magnetic resonance imaging

Experimental design

Design type	Not applicable for diffusion imaging data.
Design specifications	Not applicable for diffusion imaging data.
Behavioral performance measures	60 different behavioral scores spanning cognition, emotion and personality, from the HCP database were used for behavioral predictions. Information on behavioral scores can be found at https://www.humanconnectome.org/study/hcp-young-adult/project-protocol/behavioral-testing.

Acquisition

Imaging type(s)	Structural, Diffusion
Field strength	3 Tesla
Sequence & imaging parameters	Dataset I: Spin echo sequence, EPI, FoV=256mm, matrix 128 slice thickness=2mm, orientation:T>C-7.3>S-0.7, TE=90ms, TR:8900ms, flip angle=8 degrees.<br>Dataset S: Details of scan protocols can be found in Pestilli et al., 2014 (https://www.nature.com/articles/nmeth.3098).<br>Dataset M: Synthetic data generated using a single subject dataset from the HCP database, details for which can be found at https://www.humanconnectome.org/storage/app/media/documentation/s1200/HCP_S1200_Release_Reference_Manual.pdf.<br>Dataset H and data for behavioral predictions: Details of scan protocols can be found at https://www.humanconnectome.org/storage/app/media/documentation/s1200/HCP_S1200_Release_Reference_Manual.pdf.
Area of acquisition	Whole brain

Diffusion MRI ☒ Used ☐ Not used

Parameters	Dataset I: 64 directions (plus 2 b=0 acquisitions), single-shell (b-value=1000 s/mm2). No cardiac gating was used.<br>Dataset S: 96 directions (plus 10 b=0 acquisitions), single-shell (b-value=2000 s/mm2). No cardiac gating was used.<br>Dataset M: Synthetic data simulated from single subject from HCP database: 270 directions (plus 18 b=0 acquitions), multi-shell (b-values=1000, 2000, 3000 s/mm2).<br>Dataset H and data for behavioral predictions: 270 directions (plus 18 b=0 acquitions), multi-shell (b-values=1000, 2000, 3000 s/mm2).

Preprocessing

Preprocessing software	MRtrix3, FSL, version 5.0, FreeSurfer version 6.
Normalization	Datasets I, S, and M were not normalized. Dataset H and all scans used for behavioral prediction were normalized to the MNI template.
Normalization template	MNI template.
Noise and artifact removal	Datasets I and S: Denoising, motion, and eddy correction.<br>Dataset M: Denoising, Gibbs ringing artifact removal, motion and eddy correction, and susceptibility-induced distortion correction.<br>Dataset H and all data used for behavioral predictions: We used the minimally preprocessed data available in the HCP database. Preprocessing steps included B0 intensity normalization, susceptibility-induced distortion correction, motion and eddy correction, and gradient non-linearity correction.
Volume censoring	No volume censoring was performed in any dataset.

Statistical modeling & inference

Model type and settings

For connectome estimation using MRtrix3, the Constrained Spherical Deconvolution (CSD) model was used to estimate Fiber Orientation Distributions (FODs), followed by probabilstic tractography.
For connectome evaluation using LiFE and ReAl-LiFE the Stejskal-Tanner model was used for estimating the diffusion signal based on a structural connectome, b-values and gradient directions.

Effect(s) tested

To quantify LiFE/ReAl-LiFE model accuracy, voxelwise root mean squared error (RMSE) between the measured and predicted diffusion signals were computed.
For behavioral score prediction analyses, Bend correlation was computed between predicted and actual scores.

Specify type of analysis: ☐ Whole brain ☐ ROI-based ☒ Both

Anatomical location(s)

Anatomical segmentation was performed using FreeSurfer. The Desikan-Killiany atlas was used for all analyses.

Statistic type for inference
(See Eklund et al. 2016)

The Kolmogorov-Smirnov test and Wilcoxon signed-rand test was used to compare the voxelwise RMSE post pruning with SIFT/SIFT2, LiFE and ReAl-LiFE.
For behavioral score predictions, significantly predicted scores were identified for a range of significance levels and also using a permutation test.

Correction

For behavioral score predictions, corrections for multiple comparisons was carried out using the Benjamini-Hochberg correction.

Models & analysis

n/a	Involved in the study
☒	☐ Functional and/or effective connectivity
☒	☐ Graph analysis
☐	☒ Multivariate modeling or predictive analysis

Multivariate modeling and predictive analysis

Behavioral score predictions using structural connectivity features were carried out using Support Vector Machine (SVM) based regression model with a linear kernel. Correlation between observed and predicted scores were carried out using the Bend correlation, correcting for univariate outliers (details provided in the Methods).
