# Supplementary material for: GPU-accelerated connectome discovery at scale
Source: Nat Comput Sci. 2022 May 30;2(5):298–306. doi: 10.1038/s43588-022-00250-z (PMC10766542; doi:10.1038/s43588-022-00250-z)
Supplement: Supplementary file 5 — Data to reproduce Fig. 1. [file 43588_2022_250_MOESM5_ESM.zip › Source_Data_Figure_1/Readme.txt]

All variables in the .mat files may be converted to excel files in MATLAB as follows:load(’name_of_mat_file.mat’);xlswrite(‘name_of_excel_file.xlsx’, variable_name);
